# Bio‐Inspired Imprinting Materials for Biomedical Applications

**DOI:** 10.1002/advs.202202038

**Published:** 2022-07-31

**Authors:** Hanxu Chen, Jiahui Guo, Yu Wang, Weiliang Dong, Yuanjin Zhao, Lingyun Sun

**Affiliations:** ^1^ Department of Rheumatology and Immunology Nanjing Drum Tower Hospital School of Biological Science and Medical Engineering Southeast University Nanjing 210096 P. R. China; ^2^ State Key Laboratory of Materials‐Oriented Chemical Engineering College of Biotechnology and Pharmaceutical Engineering Nanjing Tech University Nanjing 211800 P. R. China; ^3^ Oujiang Laboratory (Zhejiang Lab for Regenerative Medicine, Vision and Brain Health) Wenzhou Institute University of Chinese Academy of Sciences Wenzhou Zhejiang 325001 P. R. China

**Keywords:** bio‐inspired, biomedical applications, molecular imprint, sensitivity, specificity

## Abstract

Inspired by the recognition mechanism of biological molecules, molecular imprinting techniques (MITs) are imparted with numerous merits like excellent stability, recognition specificity, adsorption properties, and easy synthesis processes, and thus broaden the avenues for convenient fabrication protocol of bio‐inspired molecularly imprinted polymers (MIPs) with desirable functions to satisfy the extensive demands of biomedical applications. Herein, the recent research progress made with respect to bio‐inspired imprinting materials is discussed in this review. First, the underlying mechanism and basic components of a typical molecular imprinting procedure are briefly explored. Then, emphasis is put on the introduction of diverse MITs and novel bio‐inspired imprinting materials. Following these two sections, practical applications of MIPs in the field of biomedical science are focused on. Last but not least, perspectives on the remaining challenges and future development of bio‐inspired imprinting materials are presented.

## Graphical abstract

Inspired by the recognition mechanism of biological molecules, molecular imprinting techniques are imparted with numerous merits and thus broaden the avenues for convenient fabrication protocol of bio‐inspired molecularly imprinted polymers with desirable functions to satisfy the extensive demands of biomedical applications. Herein, the recent research progress made with respect to bio‐inspired imprinting materials in this review is discussed.

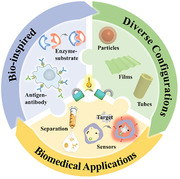

## 1 Introduction

Molecular imprinting is the technique that constructs a specific rebinding system with selective and robust recognition sites to target molecules in synthetic polymers and bionics. The origin of molecular imprinting technology (MIT) dates back to the 1930 s when Polyakov found adsorption properties of silica gel and first proposed the concept of “molecular imprinting”.^[^ ^1^ ^]^ Since then, tremendous interest has been attracted to the design, preparation, and characterization of MIT and numerous molecularly imprinted polymers (MIPs) have been gradually developed.^[^ ^2^, ^3^, ^4^, ^5^ ^]^ Inspired by the recognition interactions between the natural biological molecules, MIT is often described as an approach to forming a “key‐lock” structure based on the spatial topology, functional groups, or charge distribution between target templates and functional monomers, which contributes to recognizing and selectively rebinding template molecules from complex compounds, such as atom, ions or biological/chemical molecules, etc.^[^ ^6^, ^7^, ^8^, ^9^, ^10^ ^]^ In particular, the binding sites can be flexibly designed according to the physicochemical property of target templates, meanwhile the functional monomers and cross‐linkers can also be adjustable to endow molecularly imprinted materials with more excellent mechanical capacity or biochemistry functions. Besides, the fabrication processes can be operated under relatively simple conditions and environments. Therefore, molecular imprinting bears extensive practical value and enables the generation of materials or polymers with excellent stability, adsorption properties, and easy synthesis processes that would be difficult to obtain in other approaches.^[^ ^11^, ^12^, ^13^, ^14^ ^]^

The bio‐inspired MIPs can be fabricated by copolymerizing functional monomers and cross‐linkers around target template molecules, eventually forming highly cross‐linked polymers. The interactions between template molecules and functional monomers form robust binding sites based on covalent, noncovalent, and semi‐covalent interactions.^[^ ^15^, ^16^, ^17^ ^]^ After the removal of template molecules by elution, the unique spatial cavities of template molecules remain in the polymer matrix, which records the shape, size, charge, or chemical functionality of templates and realizes the specific recognition to target.^[^ ^18^, ^19^, ^20^ ^]^ Compared to other traditional recognition systems such as antibody‐antigen, enzyme‐substrate, and biological receptors, MIT not only mimics natural recognition mechanisms and processes, but also possesses more promising characteristics including predictable and designable structure, high chemical‐physical stability, excellent reusability, and low fabrication cost. Such features have paved the way for wider applications of MIT in biomedical fields, such as sample separation and purification, biosensing, artificial antibodies, catalysis, drug delivery, etc.^[^ ^21^, ^22^, ^23^, ^24^ ^]^ Therefore, great progress of MIT has brought bright development prospects in physical, chemical, biological, medical fields, and environmental science.

Due to the wide application prospect and promising potential of MIT, several classic reviews on MIT have been published, which mainly placed emphasis on certain basic aspects like fundamental preparation processes and characteristic applications of MIPs.^[^ ^25^, ^26^, ^27^, ^28^ ^]^ However, a comprehensive review on bio‐inspired imprinting materials about new fabrication mechanisms, working principles, and novel applications in different biomedical fields in recent years remains lacking. Thus, we believe that such a substantial and general review about all aspects of MIT would have a profound impact on this active and multidisciplinary field. Such a review could inspire scientists of different professional backgrounds to explore the development direction of MIT in various fields of science.

Here, we give an overall review of the research progress of bio‐inspired imprinting materials derived from MIT, covering the fundamentals to practical applications. First of all, we briefly introduce the fundamentals of molecular imprinting and summarize the basic mechanism and critical elements of molecularly imprinted materials. Then we focus on the classifications of diverse molecular imprinting techniques (MITs), including traditional imprinting (surface‐imprinting, nanoimprinting) and novel imprinting (multiply imprinting, stimuli‐responsive imprinting). Following these two opening sections, we mainly show concerns about the biomedical applications of bio‐inspired imprinting materials. The applications of MIT in sample separation and chemical/biological sensing are described respectively. Finally, we analyze the current status and propose the remaining challenges with future perspectives and development directions of MITs.

## 2 Fundamentals of Molecular Imprinting

**Figure 1:**
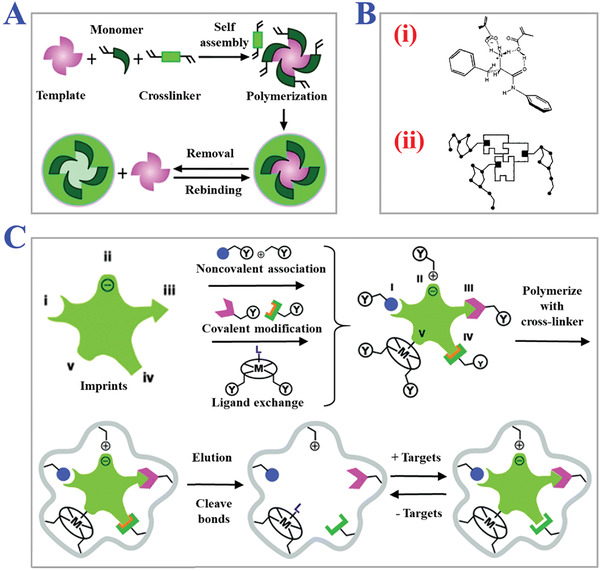
Fundamental principle of MITs. A) Schematic fabrication procedure of MIPs. Reproduced with permission.^[^ ^55^ ^]^ Copyright 2012, The Royal Society of Chemistry. B) The interaction forces between the target templates and functional monomers: i) basic interactions; ii) more complexed interactions. Reproduced with permission.^[^ ^7^ ^]^ Copyright 2012, John Wiley & Sons, Ltd. C) Several main types of molecular imprinting, including covalent imprinting, non‐covalent imprinting, semi‐covalent imprinting, electrostatic/ionic, and metal center coordination. Reproduced with permission.^[^ ^37^ ^]^ Copyright 2014, The Royal Society of Chemistry.

Fundamentals of MIT can be briefly described as MIPs, which are synthesized in the molecular imprinting process to play a key role in specific recognition functions. The interaction forces between the target templates and functional monomers are the basis of constructing recognition and rebinding sites in the network of polymer matrix (**Figure** **1**A,B).^[^ ^29^, ^30^ ^]^ Besides, the copolymerization processes including polymerization methods, reaction conditions, and introduction of assistant compounds also influence the properties and performance of final polymerized MIPs. Currently, fundamental research has been further explored for the development of novel MITs.^[^ ^31^, ^32^, ^33^, ^34^ ^]^

### 2.1 Imprinting Mechanism

The mechanism of molecular imprinting is to specifically recognize and rebind target template molecules based on the “key‐lock” structure like antibody‐antigen or enzyme‐substrate. The “lock” structure refers to the imprinting site remaining in the 3D network of polymerized matrix, and the imprinted target template molecules act as the role of “key”. The imprinting sites record the unique chemical, electronical, or structural information of target template molecules, such as functional groups, charge distribution, or spatial topological structures in MIPs, which enhance the specificity of rebinding process.^[^ ^35^, ^36^ ^]^ The fabrication of the above imprinting sites mainly relies on the robust combination and interaction between target template molecules and functional monomers, including covalent interactions, noncovalent interactions, and semi‐covalent interactions, as schemed in Figure 1C.^[^ ^37^ ^]^

#### 2.1.1 Covalent Imprinting

Covalent imprinting as a typical method has been widely used for molecular imprinting due to the precisely fixed functional groups that remained during polymerization and complete removal of target template molecules. As Wulff et al. reported, the covalent linkage formed within polymerization would be cleaved when target templates were removed.^[^ ^17^ ^]^ During rebinding process, the same covalent linkage would be reformed to specifically recognize and capture the guest target template molecules. For example, boronic acid has been demonstrated suitable for covalent imprinting.^[^ ^38^, ^39^, ^40^ ^]^ The stable trigonal boronic acid esters can realize reversible condensation which forms covalent boronic acid imprinting with high affinity to compounds containing diol groups. Other covalent imprinting methods involving acetal/ketal, Schiff's base reaction, chelate complex, amide, and so on have also been widely applied for covalent imprinting.^[^ ^41^, ^42^, ^43^, ^44^ ^]^ However, it is difficult to realize the thermodynamic equilibrium of strong covalent interaction, which would inevitably cause slow dissociation and rebinding process. In addition, the suitable eversible condensation reactions are usually confined within the above types, which is difficult to satisfy the diverse needs of imprinting different template molecules, further limiting the wider applications of covalent imprinting.

#### 2.1.2 Noncovalent Imprinting

Different from covalent imprinting, noncovalent imprinting constructs the binding sites between target template molecules and functional monomers with the help of noncovalent interactions involving ionic interactions, dipole‐dipole interactions, hydrogen‐bond formation, van der Waals forces, and electrostatic interactions, etc. In noncovalent imprinting, the noncovalent interaction between functional monomers and template molecules can easily reach thermodynamic equilibrium in a polymerization environment. Mosbach et al. proposed that the usages of ligand functional monomers tended to be much larger than template molecules, which ensured the occurrence of rapid and sufficient noncovalent reactions.^[^ ^16^ ^]^ Benefitting from the advantages of the rapid dissociating and rebinding process, noncovalent imprinting has been considered as an important synthesis strategy to produce MIPs on a large scale. Moreover, the operation processes of noncovalent imprinting including reaction medium environment, reaction conditions, and catalyst systems are relatively easy compared with covalent imprinting. One typical noncovalent imprinting is based on the noncovalent binding between methacrylic acid (MAA) and amide compounds in nonpolar solvents.^[^ ^45^, ^46^, ^47^ ^]^ The oxhydryl groups (‐OH) in MAA tend to form strong electrostatic interaction and hydrogen‐bond to oxygen atom in amides. These two noncovalent interactions cooperate to enhance the specificity due to the high selectivity cavities formed in MIPs. Although noncovalent imprinting has been extensively used, there still exist many shortcomings. The massive usages of functional monomers during polymerization will form excessive and non‐specific binding groups after the removal of template molecules, which hinder the formation and influence the function of MIPs (for example, catalysts).^[^ ^48^, ^49^ ^]^ Besides, noncovalent interactions are easily disrupted by the presence of water in the reaction solvent environment. Therefore, more new and robust binding groups are highly desired to explore the applications of noncovalent imprinting.

#### 2.1.3 Semi‐Covalent Imprinting

Semi‐covalent imprinting has merged as a novel MIT that combines the advantages of covalent imprinting and noncovalent imprinting, involving stability, durability, and rapid rebinding. In semi‐covalent imprinting, the template molecules combine with functional monomers based on covalent interactions while the MIPs rebind the target molecules through noncovalent interactions.^[^ ^50^ ^]^ There are two variations in the semi‐covalent approach which can be distinguished by the connection between the template and the monomer, including direct connection or connection with spacer groups. The covalent imprinting step of forming template‐monomers complex during polymerization can overcome the disadvantages of excessive non‐specific binding groups through noncovalent imprinting. Meanwhile, the rebinding processes mainly rely on noncovalent interaction with guest template molecules which can dramatically improve the rebinding sensitivity and efficiency.^[^ ^51^, ^52^, ^53^ ^]^ The resultant MIPs tend to possess much higher specificity and sensitivity to target template molecules than those fabricated by single imprinting techniques. Therefore, semi‐covalent imprinting has been considered as a promising method to synthesize more homogeneous imprints, imparting the imprinting process with dynamic self‐correcting capabilities and fabricating more universal artificial antibodies. Zimmerman et al. have reviewed the presentative characteristics of perfect MIPs and these excellent advances can be made based on the development of semi‐covalent imprinting.^[^ ^5^ ^]^

Traditional mechanisms of molecular imprinting have been thoroughly investigated, and novel mechanisms are still under exploration for the better development of MIPs. Both advantages and disadvantages of several types of MIT have been reported.^[^ ^54^, ^55^, ^56^ ^]^ Although there exist more and more types of molecular imprinting, the researches on mechanisms are still fundamental. The exploration about principles and mechanisms of specific recognition and rebinding to target consistently influence the development directions of MIT. Generally, the types of molecular imprinting should be selected according to the suitable mechanism, desired properties, and functions of resultant MIPs.

### 2.2 Fabrication Procedure

Traditional molecular imprinting fabrication procedures tend to involve the following steps (Figure 1A). First, appropriate functional monomers with robust binding capability to target template molecules should be chosen based on the physical‐chemical properties and several interaction forces. Then with the help of cross‐linkers, a complementary complex of template‐monomers is fabricated by performing a copolymerization reaction. In this step, some other assistant compounds such as initiators or porogenic solvents can be added to promote polymerization or the formation of special porous structures. Finally, target template molecules are removed, leaving the imprinting sites in the network of polymer matrix and forming desired MIPs eventually. Many factors in the fabrication procedure would influence the polymerization reaction, such as the usage ratio of compounds, chemical accessories, solvent environment, reaction temperature, polymerization time, etc.^[^ ^57^ ^]^ In this section, we will discuss the essential elements above and their effects on the imprinting polymerization process.

#### 2.2.1 Reagents Preparation

**Figure 2:**
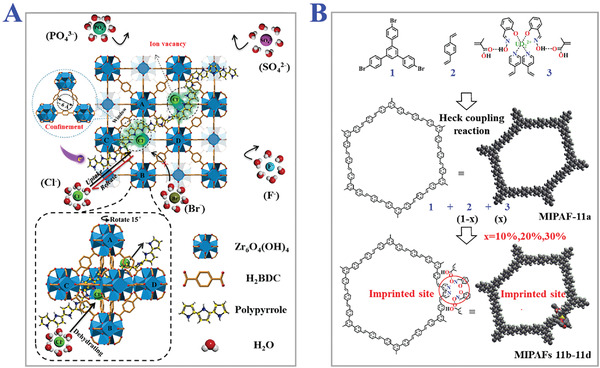
Constructions of inorganic ions‐functional monomers compounds in different MIPs frameworks. A) Threaded chlorine ions‐imprints in metal‐organic frameworks. Reproduced with permission.^[^ ^61^ ^]^ Copyright 2021, Elsevier B.V. B) Uranium ions imprint in porous aromatic frameworks. Reproduced with permission.^[^ ^62^ ^]^ Copyright 2018, Wiley‐VCH GmbH.

**Figure 3:**
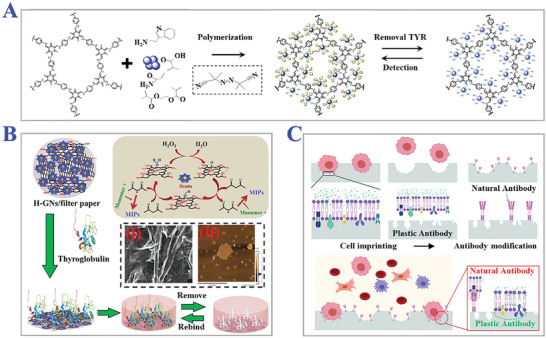
Different classifications of target templates. A) Organic molecules like tryptamine as target template. Reproduced with permission.^[^ ^67^ ^]^ Copyright 2019, Elsevier B.V. B) Biomacromolecule like thyroglobulin (Tg) as template analytes. i) SEM and ii) AFM images of Tg‐imprinted MIPs paper. Reproduced with permission.^[^ ^68^ ^]^ Copyright 2019, Elsevier B.V. C) Living materials like circulating tumor cells (CTCs) as templates. Reproduced with permission.^[^ ^69^ ^]^ Copyright 2021, Wiley‐VCH GmbH.

Typical molecular imprinting protocol comprises of three basic reagents, including template molecules, functional monomers, and cross‐linkers. The goal of molecular imprinting is to fabricate MIPs with high affinity and specificity to target template molecules. Such affinity and specificity are dramatically influenced by the selection of appropriate reagents. Generally, ideal template molecules should satisfy the following requirements.^[^ ^58^ ^]^ First, the target as a template should contain rich functional groups to combine with functional monomers, and the binding in template‐monomer complex should not prevent polymerization or be disrupted by polymerization reaction. In addition, the template molecule itself should possess stable chemical properties and avoid resolving or inactivation during polymerization. So far, commonly used template molecules can be mainly divided into four types, including inorganic ions, organic molecules, biomacromolecules, and living biomaterials.^[^ ^59^, ^60^ ^]^ Inorganic ions, especially metal ions, have been applied as templates to fabricate ion‐imprinted polymers for the detection and removal of toxic metal ions in environmental pollutants. Ligand complexes are used to assemble with metal ions to overcome the weak selectivity due to the similar ionic radii and charge distribution, as shown in **Figure** **2**.^[^ ^61^, ^62^ ^]^ For example, Chen et al. designed Hg^2+^ ion‐imprinted polymers (IIPs) by a sol‐gel process for mercury speciation analysis in environmental and biological samples.^[^ ^63^ ^]^ They synthesized dithizone chelated with Hg^2+^ as templates to copolymerize with 3‐aminopropyltriethoxysilane as a functional monomer. The resultant IIPs showed higher affinity and selectivity to Hg^2+^ than organic Hg and other metal ions. Biomacromolecule imprints such as proteins also arouse the great interest of researchers due to the urgent need of controlling biological form and functions for better biomedical applications.^[^ ^64^, ^65^ ^]^ Bossi and colleagues first proposed the concept of “gate effect”, which referred to the properties of transporting proteins by MIPs under external conditions based on the adjustable pore sizes in polymeric matrix.^[^ ^66^ ^]^ They imprinted hydrophilic poly(acrylamide) membranes with proteins, and the pore sizes could be influenced under electrophoresis. The “gate effect” of MIPs has been widely used to selectively translate proteins for proteomic analysis. Additionally, organic molecules (for example, glucose, pharmaceuticals, etc.), macromolecules (for example, proteins, polysaccharides, etc.), living biomaterials (for example, cells, viruses, and bacteria, etc.) have also been reported for practical applications of MIT, as presented in **Figure** **3**.^[^ ^67^, ^68^, ^69^ ^]^

Functional monomers are commonly used to form template‐monomer complexes before polymerization by covalent or noncovalent interactions. The monomers usually contain recognition units and polymerization units. The recognition unit provides functional groups to interact and combine with template molecules, while the polymerization unit realizes the construction of a polymeric matrix with the help of cross‐linkers under certain conditions. The template‐monomer complexes have similar structures like antibody‐antigen or enzyme‐substrate substances, and the binding strength directly influences the affinity and selectivity of resultant MIPs. Hence, monomers with more stable and suitable functional groups should be chosen to better match with template molecules. Karim and coworkers have reviewed diverse strategies for the selection of effective functional monomers, including Morphological characteristics, Nuclear Magnetic Resonance (NMR), Fourier Transform Infrared Spectroscopy (FTIR), UV spectroscopic titrations, Computer simulation, Combinatorial screening, and so on.^[^ ^70^, ^71^ ^]^ Mizaikoff and colleagues have used molecular dynamics simulations and ^1^H NMR spectroscopy to select suitable monomers for 17*β*‐estradiol among 18 candidate monomers.^[^ ^72^ ^]^ Current functional monomers can be classified into three categories, respectively corresponding to covalent, noncovalent, and semi‐covalent imprinting. Among these monomers, MAA is considered as a universe choice because of the ability to form a stable hydrogen bond with templates. Shimizu et al. studied the influence of MAA dimerization during imprinting process.^[^ ^73^ ^]^ They designed MAA‐based MIPs imprinted with nucleobases and ethyl adeine‐9‐acetate (EA9A) severally. It was demonstrated that the large pores distributed in MIPs by high molar fractions of MAA actually could enhance the rebinding of template molecules. Apart from traditional functional monomers, some monomers with special properties and structures have also shown potential in preparing MIPs for more bio/chemical needs. For instance, Zanin's group imprinted cholesterol with *β*‐cyclodextrins (*β*‐CDs) as monomers that possessed a hydrophilic exterior and hydrophobic non‐polar cavity in the center.^[^ ^74^ ^]^ The amphipathy contributed to forming an inclusion complex with a variety of templates, and the final *β*‐CD‐based MIPs achieved adsorption capacity to target cholesterol. In particular, a critical point deserving great attention in the synthesis process is the molar ratios between the templates and monomers. Too low molar ratio of monomers will cause few binding sites in the network or even hinder the polymerization, while a too high molar ratio will form excessive non‐specific binding sites and weaken the specific recognition of templates. Hence the suitable ratio should be characterized to satisfy the best reaction conditions.

Cross‐linkers are introduced into the imprinting process to help fit functional monomers around template molecules rigidly and form highly cross‐linked polymeric matrix, taking advantage of that the morphology and mechanical stability of MIPs can be controlled after the removal of templates molecules. Notably, the most important function of cross‐linkers is to stabilize complementary structures of topological shape and chemical functionality in 3D MIPs. Such cross‐linkers mainly include tetraethoxysilane (TEOS), glycidilmethacrylate (GMA), 3‐aminopropyltriethoxysilane (APTES), ethylene glycol dimethacrylate (EGDMA), etc.^[^ ^75^, ^76^ ^]^ Similar to monomers, the usages of cross‐linkers also influence the properties of resultant MIPs, which means too low usage of cross‐linkers would reduce the mechanical strength to maintain the imprinting structure. Row et al. reported that high cross‐linked ratios could be preferred to access permanently porous materials where the functional groups possessed optimal distribution and spatial configuration for better rebinding of templates.^[^ ^77^ ^]^ However, too high usage of cross‐linkers will reduce active binding sites for recognition of targets, due to the excessively compact space where a few sites can be exposed as receptors. Thus, basic cross‐linkers ratio should be optimized previously to ensure the best polymerization. Other assistant compounds such as initiators and porogenic solvents have also been investigated to synthesize MIPs with more special structures or properties. Initiators need to correspond to the type of polymerization including heat, light, chemical, electronic, and so on. A typical initiator is azobisisobutyronitrile (AIBN), which possesses the ability to trigger and control the polymerization of methylmethacrylate towards poly(methylmethacrylate) (PMMA) under both photolysis (UV) and thermolysis.^[^ ^78^, ^79^ ^]^ Porogenic solvents with relatively low polarity and high solubility phase tend to separate later during polymerization, forming small pores and great surface areas, which provide MIPs with sufficient adsorption and rebinding area. Sellergren and Shea demonstrated that the use of porogen MeCN with poor hydrogen bonding capacity promoted the selectivity and rebinding of L‐PheNHPh.^[^ ^80^ ^]^ Remarkably, due to the phenomenon of “solvent memory”, MIPs fabricated within organic porogenic solvents showed poor efficiency in aqueous solvent.

#### 2.2.2 Synthesis Methods

**Figure 4:**
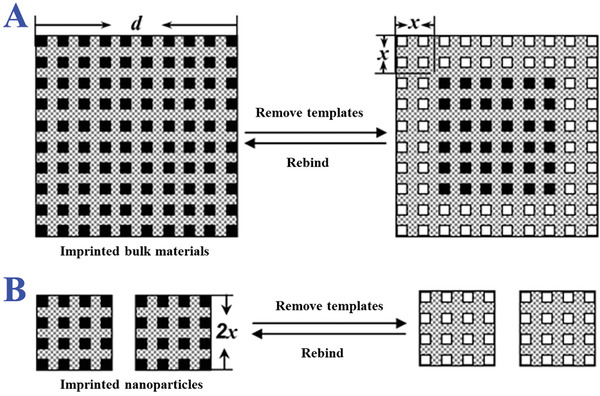
Scheme of the distribution of the binding sites in the A) imprinted bulk materials and B) nanoparticles. The unnecessary heterogeneous binding sites would hinder the specific binding in imprinted bulk materials especially. Reproduced with permission.^[^ ^84^ ^]^ Copyright 2007, American Chemical Society.

MIPs fabricated by traditional synthesis methods are generally comprised of molecularly imprinted particles and monoliths. The monoliths face complex disposing processes like crushing, sieving, and grounding, which bring great challenges for further applications. Here we mainly review the common synthesis methods of preparing molecularly imprinted particles including bulk polymerization, suspension polymerization, seed polymerization, and precipitation polymerization. Bulk polymerization has attractive advantages of rapidity, simplicity, purity, and easy operation conditions for preparing molecular imprinting particles and this method has become a popular strategy for mass production.^[^ ^81^, ^82^ ^]^ Baggiani et al. synthesized several carbamate‐imprinted polymers with different functional monomers and porogenic solvents by basic bulk polymerization and demonstrated the enhanced selectivity features.^[^ ^83^ ^]^ However, resultant MIPs of this method usually mix with monoliths, and the following crushing and sieving operation inevitably results in irregular particle sizes and loss of rebinding affinity, as illustrated in **Figure** **4**.^[^ ^84^ ^]^ Templates located within *x*‐nanometers from the surface were assumed to be able to remove in bulk materials with scale *d*. The effective volume of resultant imprinted sites can be calculated by *d* ^3^ − (*d* − 2*x*)^3^. Under normal conditions, *x* value is small for bulk materials regardless of porogens or solvents. If imprinted materials with the same size were prepared with a nanostructure scale of 2*x* nm, all templates could be fully removed from polymer matrix and these binding sites were effective for target species. Besides, bulk polymerization can only impart the final MIPs with heterogeneous binding sites, which confines wider applications of MIPs.

**Figure 5:**
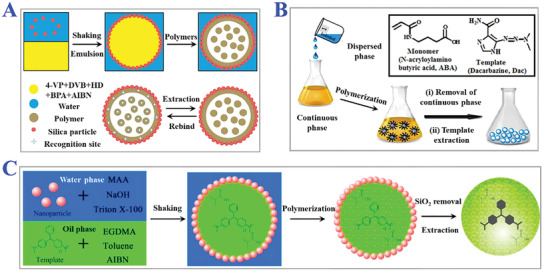
Examples of suspension polymerization. A) The fabrication procedure of MIPs microrattles bearing multicore constructions induced by suspension polymerization. Reproduced with permission.^[^ ^85^ ^]^ Copyright 2017, Elsevier B.V. B) The synthesis of MIPs nanospheres for dacarbazine via inverse suspension polymerization method. Reproduced with permission.^[^ ^86^ ^]^ Copyright 2017, Elsevier B.V. C) Malachite green‐imprinted MIPs microspheres on the basis of pickering emulsion droplets. Reproduced under a Creative Commons (CC‐BY) license.^[^ ^87^ ^]^ Copyright 2017, The authors. Licensee MDPI, Basel, Switzerland.

To cover the shortcomings of bulk polymerization, suspension polymerization has been chosen as an alternative with the advantages of reproducible resultants, high yields, monodispersity, and regular microspheres, as presented in **Figure** **5**.^[^ ^85^, ^86^, ^87^ ^]^ Suspension polymerization provides liquid continuous phase to suspend the droplet of pre‐polymerization mixtures, namely oil in water (O/W) droplets and water in oil (W/O) droplets, which contain template molecules, functional monomers, cross‐linkers, and initiators. To ensure the stability of droplets, stabilizers or surfactants are added in aqueous and oil phase to reduce surface tension between the liquid interface. The sizes of droplets differ on a large scale according to the different reaction conditions, which could be disturbed by the dispersing medium. Ersoz and coworkers proposed MIPs of L‐histidine (L‐His) for high‐performance liquid chromatography (HPLC) stationary phases based on emulsion suspension polymerization.^[^ ^88^ ^]^ The droplets comprised of N‐methacryloyl‐L‐His‐copper(II)‐L‐His, EGDMA, and crosslinkers were suspended in water. The resultant L‐His imprinted polymers were demonstrated suitable for biochromatography applications. Water, commonly used as a continuous phase, tends to hinder hydrophilic and electrostatic interactions between templates and monomers, which limits the suspension polymerization application in noncovalent imprinting. Some other continuous phases like liquid perfluorocarbon and mineral oil have been developed to avoid multi‐step swelling process caused by water.^[^ ^89^, ^90^ ^]^ Such mediums possess stable chemical inertness, which are immiscible with varieties of organic solvents and MIP reagents. On the contrary, porogens including chloroform, dichloromethane, and toluene are both miscible with the above mediums to form porous structures well during polymerization. Mosbach et al. employed acrylate perfluorocarbons as a continuous phase to suspend emulsions of noncovalent imprinting mixtures with the help of surface‐active poly‐(oxyethylene) containing fluorinated units and ester groups.^[^ ^91^ ^]^ The resultant tert‐butoxycarbonyl‐L‐phenylalanine (Boc‐L‐Phe) imprinted polymer beads gave low back pressure and rapid diffusion, giving good separation at high flow rates of HPLC tests. However, special fluorinated surfactants are necessary for stable suspension of pre‐polymerization emulsions in different mediums, which limits the practicality of this method.

**Figure 6:**
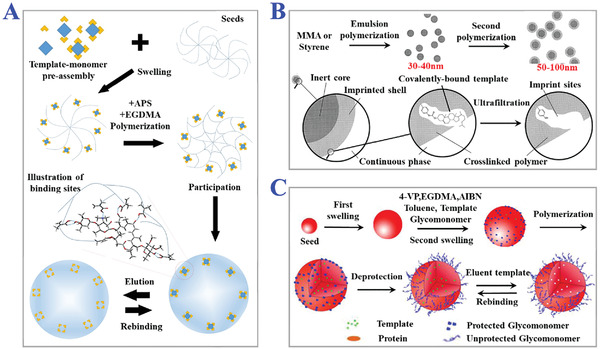
The applications of seed polymerization. A) The scheme of seeds‐assisting polymerization of micron‐sized MIPs particles for purification of tylosin form broth. Reproduced with permission.^[^ ^92^ ^]^ Copyright 2020, Elsevier Ltd. B) The process of two‐stage seed polymerization for fabrication of submicron MIPs particles with cholesterol‐imprinted shells. Reproduced with permission.^[^ ^93^ ^]^ Copyright 2000, Wiley‐VCH. C) Modifying uniform‐sized glycol‐MIPs with surface hydrophilic sugar moiety for detection of phenobarbital imprints via two‐step swelling seed polymerization. Reproduced with permission.^[^ ^94^ ^]^ Copyright 2011, Elsevier Ltd.

Seed polymerization is also named as a multi‐step swelling method to produce comparatively monodispersed MIP beads. During typical seed polymerization, seed particles with uniform sizes are chosen as templates which are swollen afterwards by emulsion droplets with activating solvent and prepolymerization mixtures. The swelling processes are terminated by the following polymerization to eventually form desired monodispersed particles. We enumerated several practical applications of seed polymerization in **Figure** **6**.^[^ ^92^, ^93^, ^94^ ^]^ It was worth noting that the surface of resultant MIPs could be easily modified in situ based on seed polymerization by addition and dispersion method. The addition method meant adding polar monomers like glycerol dimethacrylate (GDMA) and glycerol monomethacrylate (GMMA) after the initiation step to modify the outer surface and macropores (>50 nm). The dispersion method meant dispersing MIPs in GDMA and GMMA mixture solution to modify the inner micropores (1–50 nm). More importantly, these two methods will not influence the structures and morphology of micropores (<1 nm). Sanbe and colleagues designed restricted access media‐imprinted MIPs (RAM‐MIP) which were modified with a hydrophilic external layer by directly adding 1:1 mixture of GDMA and GMMA to MIPs.^[^ ^95^ ^]^ The RAM‐MIP showed excellent recognition of propranolol (PRP) and other *β*‐blockers due to the critical hydrophilic modification. Whereas, the multi‐step process is greatly tedious and time‐consuming including the growth of seed particles, swelling process, and modification procedure. Besides, the aqueous phase will interfere with imprinting mixtures, influence the interactions between templates and monomers, and thus lead to low selectivity and affinity finally.

**Figure 7:**
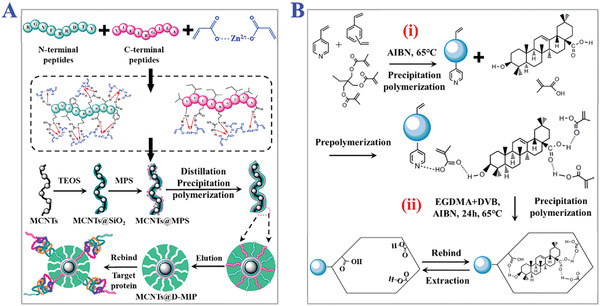
The applications of precipitation polymerization. A) The synthesis process of dual‐templates MIPs coated on magnetic carbon nanotubes via metal‐chelation and distillation‐precipitation polymerization for recognition of PSA. Reproduced with permission.^[^ ^99^ ^]^ Copyright 2018, Elsevier B.V. B) Schematic illustration of two‐step precipitation polymerization procedure. i) The first step is to form copolymeric microsphere, and ii) the second step is to modify such microspheres with oleanolic acid‐imprinted MIPs layer. Reproduced with permission.^[^ ^100^ ^]^ Copyright 2018, Wiley‐VCH.

Precipitation polymerization is another promising polymerization method to produce MIP particles with more spherical surfaces and uniform sizes.^[^ ^96^, ^97^, ^98^ ^]^ In the precipitation system, the growing polymer chains are insoluble with monomers, or monomer and initiators are soluble with the medium while the polymer chains are not soluble, both of which eventually lead to the precipitation of formed polymer out of the reaction system. Then the polymers undergo washing and centrifugation operations to obtain resultant MIPs. Compared to the simple operations of traditional bulk polymerization, complex solvents environment and demanding reaction conditions are necessary for precipitation polymerization. Some other applications of precipitation polymerization have been generalized in **Figure** **7**.^[^ ^99^, ^100^ ^]^ In general, the diameters of MIP beads prepared by precipitation polymerization range from one or a few hundred nanometers. Zourob and his colleagues designed a spiral micro‐reactor for preparing uniform MIP beads in mineral oil with no expensive reagents or equipment, which broadened the applications of precipitation polymerization.^[^ ^101^ ^]^ By changing the reaction conditions like polymerization temperature and stirring speed, the MIP size could be controlled from nanoparticles to microbeads, and the ideal recognition affinity could be remained. Ye and coworkers investigated the precise control of particle sizes by varying the ratio of two different cross‐linkers (DVB and TRIM).^[^ ^102^ ^]^ These advantages impart precipitation polymerization with great potential for wider applications of solid phase extraction (SPE). Marcé et al. synthesized two different MIPs microspheres with well‐fined particle sizes and distribution.^[^ ^103^ ^]^ They described the subsequent use in MISPE protocols for selective extraction of carbamazepine and oxcarbazepine from human urine.

### 2.3 Characterization Methods

Many analytical techniques have been used to characterize the molecular imprinting processes and reveal the particular properties of MIPs. The surface and inner morphologies of MIPs are commonly investigated by scanning electron microscopy (SEM) and transmission electron microscopy (TEM). Atomic force microscopy (AFM) and fluorescence microscopy have also played important roles in morphology characterization. In addition, Brunauer–Emmett–Teller (BET) analysis can measure the specific surface areas and pore sizes of the MIPs via nitrogen adsorption experiments.^[^ ^104^, ^105^, ^106^ ^]^ Apart from the basic morphography characterization, increasing studies about the concrete materials, chemical elements, and interaction mechanisms have been implying new trends in characterization methods. X‐ray photoelectron spectroscopy (XPS) is a prominent method to study the chemical composition and structure of MIP materials. Huck et al. carried out XPS analysis including depth profiling and electronic mapping of N‐isopropylacrylamide (NIPAM) patterned polymer brushes which were synthesized by surface‐initiated polymerization.^[^ ^107^ ^]^ Besides, FTIR spectra, UV–vis spectroscopy, and NMR are considered as useful tools to characterize certain functional groups and interactions between templates and monomers.^[^ ^108^, ^109^ ^]^ The conformational information of MIPs always relates to the functions and properties, and even slight changes can cause the loss of desired functions or the influence of some properties. Hence, quartz crystal microbalance (QCM), ellipsometry, and scanning probe microscopy have been proposed to dynamically monitor conformational changes of MIPs. Li and coworkers fabricated PNIPAM‐co‐MBAA by radical polymerization, and they used scanning probe microscopy to investigate the behavior of polymer chains in the function of temperature.^[^ ^110^ ^]^ Some new methods have been developed to obtain information about some special properties of MIPs. For example, thermogravimetric analysis (TGA) is used to examine thermal stability, and vibrating sample magnetometer (VSM) is chosen to analyze magnetic properties of magnetic MIPs. Electrochemical methods like cyclic voltammetry (CV) and electrochemical impedance spectroscopy (EIS) are also applied to characterize charge distribution, resistance, and capacitance of MIPs, which contribute to studying the swelling and collapse process caused by ions exchange and ionic strength changes.^[^ ^111^, ^112^, ^113^ ^]^

## 3 Classifies of Molecular Imprinting Technologies (MITs)

The traditional polymerization methods have been thoroughly investigated and widely used in molecular imprinting processes. The superiority of resultant MIPs has been exploited fully in certain fields. However, the MIPs fabricated by these typical MITs still face many issues such as difficulty in finding suitable groups of templates and monomers, weak recognition and rebinding capacity, incompatibility in different liquid media, and uncontrollable stability, which severely limited the wider applications of MIPs.^[^ ^114^, ^115^ ^]^ Researchers have placed great interest in the improvement of MIPs. As a result, some novel MITs have been developed to cope with the dilemma based on the traditional molecular imprinting procedures. The classifications, innovation advantages, and applications of these smart MITs will be introduced in this section.

### 3.1 Surface Imprinting Technology

**Figure 8:**
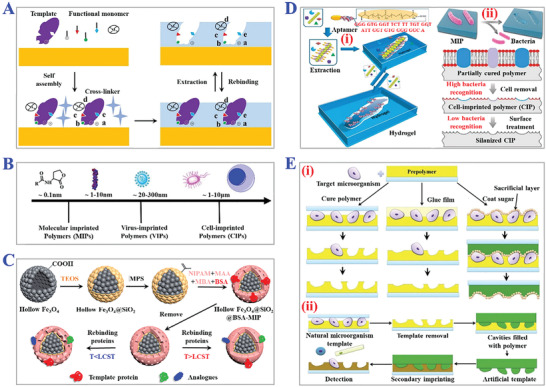
The fundamental principle and practical applications of surface imprinting technology. A) The procedure of typical surface imprinting technology. B) Spatial sizes distribution of targets templates of surface imprinting. A,B) Reproduced under the terms of the Creative Commons CC‐BY license.^[^ ^116^ ^]^ Copyright 2020, The authors. Licensee MDPI, Basel, Switzerland. C) Fabrication scheme of thermo‐sensitive MIPs with surface imprints of BSA. Reproduced with permission.^[^ ^117^ ^]^ Copyright 2019, Elsevier B.V. D) Preparation process of i) virus‐sensitive MIPs hydrogel and ii) cell‐imprinted MIPs for bacteria cells. Reproduced with permission.^[^ ^119^ ^]^ Copyright 2014, Wiley‐VCH. E) Schemes of three types of i) direct and ii) indirect micro‐contact surface imprinting procedure of target microorganism. Reproduced with permission.^[^ ^120^ ^]^ Copyright 2018, Elsevier B.V.

In brief, surface imprinting technology means fabricating MIPs by immobilizing template molecules on the surface of supporting substrates or materials, and then the polymerization processes happen subsequently on the basis of the above surface. During typical MITs procedure, the complete removal of templates is quite difficult due to the high cross‐linking structures of polymers, leading to low recognition capability. Fortunately, this problem can be well solved by introducing polymerization on the surface, which builds more effective recognition sites to realize fast mass transfer, good synthesis reproducibility, and template‐monomer binding kinetics. In particular, surface imprinting technology is considered as promising MIT for imprinting macromolecules like pharmaceuticals, proteins, cells, and viruses because their large sizes could prohibit dissociation and rebinding, as shown in **Figure** **8**.^[^ ^116^, ^117^, ^118^, ^119^, ^120^ ^]^ Zhang et al. employed surface imprinting technique to fabricate hierarchical proteins imprinted polymers by grafting porcine serum albumin (PSA) on the surface of silica microspheres.^[^ ^121^ ^]^ They realized selective depletion of human serum albumin (HSA) from human serum and explored the applications of MIPs for proteome study. Many spherical particles have been chosen as a suitable supporting substrate for surface imprinting technology, such as magnetic nanoparticles (MNPs), polystyrene beads, quantum dots (QDs), and silica gel.^[^ ^122^, ^123^ ^]^ For example, Fe_3_O_4_ MNPs are potential substrates with magnetic properties and direct purification for sample separation. The MIP‐coated Fe_3_O_4_ MNPs synthesis procedures usually contain three consecutive steps: fabrication of Fe_3_O_4_ MNPs; modification of hydrophobic MNPs surface with poly(vinyl alcohol), TEOS or ethylene glycol; surface imprinting of MNPs. Chen and coworkers designed MIP‐coated Fe_3_O_4_ MNPs for recognition and separation of estrone by semi‐covalent imprinting method in 2009.^[^ ^124^ ^]^ They prepared Fe_3_O_4_ MNPs by coprecipitation method and used TEOS to form silica shells on MNPs surface by sol‐gel process. The Fe_3_O_4_@SiO_2_ MNPs were then imprinted with estrone by simple thermal reaction to obtain the desired MIPs. The final estrone‐imprinted polymer coating Fe_3_O_4_ magnetic hybrid nanoparticles had much higher specific recognition and saturation magnetization. However, there still exist some challenges for surface imprinting technology.^[^ ^125^, ^126^ ^]^ In general, the efficiency and stability of copolymerization mainly depend on the reactive sites and compatibility of MIPs reagents on the surface of supporting substrates. The surface areas of supporting substrates for immobilization of templates inevitably limit the amount of resultant molecular imprints in MIPs for recognition and rebinding. Besides, the selection of monomers with multiply functional groups and good compatibility is critical for the successful preparation of MIP layer on the surface.

### 3.2 Nanoimprinting Technology

**Figure 9:**
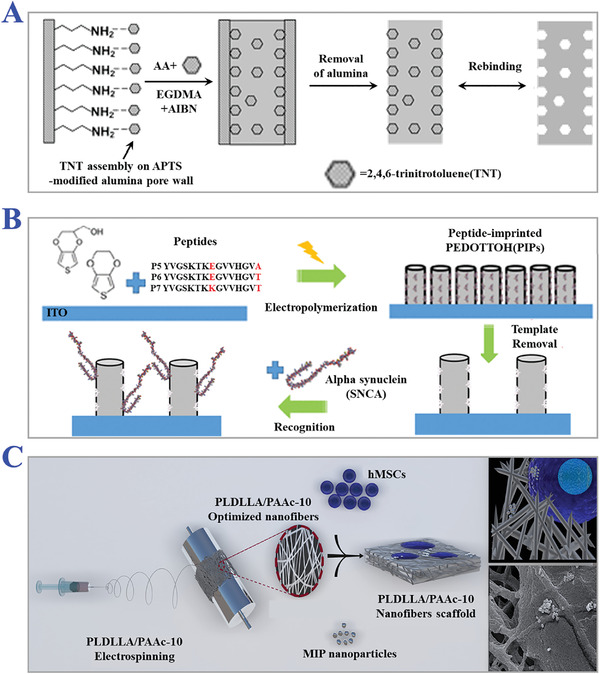
The applications of nanoimprinting technology. A) Scheme of the assembly of TNT on alumina pore walls modified with APTS, and the subsequent preparation of TNT‐imprinted nanowires with numerous imprinted sites. Reproduced with permission.^[^ ^127^ ^]^ Copyright 2006, American Chemical Society. B) Preparation of peptide‐imprinted poly(hydroxy 3,4‐ethylenedioxythiophene)‐coated nanotubes for detection of *α* synuclein in human brain organoids. Reproduced with permission.^[^ ^128^ ^]^ Copyright 2020, American Chemical Society. C) Scheme of fabrication of MIPs nanofibrous (poly(L‐lactide‐co‐D,L‐lactide)/poly(acrylic acid)) (PLDLLA/PAA) scaffolds integrated with dexamethasone‐loaded molecularly imprinted nanoparticles. Reproduced with permission.^[^ ^129^ ^]^ Copyright 2020, Elsevier B.V.

Nanoimprinting technology is a revolutionary method combining MIT with nanotechnology and nanomaterials, which fabricated MIP with nanostructures (NMIPs) like nanoparticles, fibers, nanowires, nanotubes, hybrid nanocomposites, etc., as presented in **Figure** **9**.^[^ ^127^, ^128^, ^129^, ^130^ ^]^ Different from bulk MIPs, NMIPs possess higher surface‐to‐volume ratio, thus providing easier‐accessed imprinted cavities for recognition and binding kinetics of templates. Meanwhile, almost all binding sites of NMIPs show similar affinity and specific selection to target templates. Wang et al. proposed a surface monomer‐directing strategy for highly dense imprinting of 2,4,6‐trinitrotoluene (TNT) at the surface of silica nanoparticles based on molecular nanoimprinting technology.^[^ ^84^ ^]^ They built models and analyzed the distribution of effective binding sites in bulk polymers and NMIPs respectively. It was demonstrated that nanostructured imprinted materials improved the binding capacity, kinetics, and site accessibility. In addition, MIP nanoparticles could keep stable in a certain solution, endowing them with precise manufacturing control ability, which showed a wide application prospect in drug delivery assay, capillary electrophoresis (CE), sensors, sample separation, and so on. Ciardelli and his colleagues fabricated imprinted P(MMA‐co‐MAA) nanospheres for biomedical applications such as drug release via precipitation from diluted monomer solutions.^[^ ^131^ ^]^ These acrylic polymeric nanospheres showed a comparable superiority in stable release and recognition of molecules of clinical interest. However, the fabrication procedures of NMIPs are inconvenient due to the demand for precise shape, homogeneous size, degree of crosslinking, and stable template‐monomer interactions. Choosing suitable protocols is very important for the production of NMIPs with excellent performances. Commonly employed synthetic strategies mainly involve precipitation polymerization, mini‐/micro‐emulsion polymerization, thiol ligand capping method, atom transfer radical polymerization (ATRP), reversible addition‐fragmentation chain transfer polymerization (RAFT), etc.^[^ ^132^, ^133^, ^134^ ^]^ These methods are demonstrated efficient to create NMIPs as substitute enzyme mimics and artificial antibodies. Sellergren and coworkers designed thin films composite beads of L‐phenylalanine anilide‐NMIPs by RAFT polymerization.^[^ ^135^ ^]^ They used 2‐phenylprop‐2‐yl‐dithiobenzoate as a chain transfer agent and the RAFT mediation allowed efficient control of the grafting process while prohibiting the formation of visible gel. The resultant NMIPs were able to act as chiral stationary phases with high selectivity, which could be applied for the separation of template racemates and structurally analogous racemates.

### 3.3 Multi‐Template Imprinting Technology

**Figure 10:**
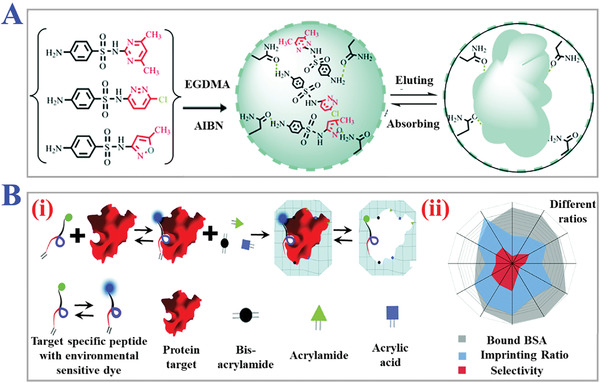
The applications of multi‐reagents imprinting technology. A) Schematic diagram of ultra‐durable, multi‐template MIPs for ultrasensitive monitoring and multicomponent quantification of trace sulfa antibiotics. Reproduced with permission.^[^ ^287^ ^]^ Copyright 2021, The Royal Society of Chemistry. B) Synthesis approach of hydro‐MIPs assembled with peptide multi‐functional monomers blocks (acrylamide, AA, and methylene bis‐acrylamide) for detection of protein (i), and the radar plot of the MIP parameters of hydro‐MIPs (ii). Reproduced with permission.^[^ ^140^ ^]^ Copyright 2018, Royal Society of Chemistry, Creative Commons Attribution‐NonCommercial 3.0 Unported License.

During traditional MIT procedures, single kind of template molecule is selected for the preparation of MIPs in most cases. In order to realize simultaneous recognition and rebinding of several targets, a feasible strategy is to prepare one MIP compound for each corresponding target template, which ensures the recognition specificity. However, different templates require individual polymerization protocols and reagents, so this strategy is relatively high‐cost and complex. Multi‐template imprinting technology, integrating binding sites of multiple templates in a single MIP, has been reported as a compositive strategy to widen the applications of MIPs, as presented in **Figure** **10**A.^[^ ^136^ ^]^ The concept of double‐template imprinting was first proposed by Screenivasan's group in 1999 to seek synthetic polymer with more than one component binding site and a high degree of selectivity to sensor elements.^[^ ^137^ ^]^ They addressed 2‐hydroxy ethyl methacrylate (HEMA)‐based MIPs with two model templates namely salicylic acid and hydrocortisone. Through the study of the participation of hydrogen bonding and uptake amounts of templates, the feasibility of imparting multiple recognition sites in a single synthetic polymer was eventually proved. Recently, multiple‐template imprinting MIPs involving three or more templates have been developed. Krupadam et al. fabricated multi‐template MIPs simultaneously imprinted with three p‐type pharmaceutical chemicals (nicotine, epinephrine, and physostigmine) for selective adsorption of multiple targets from water and wastewaters.^[^ ^138^ ^]^ The resultant MIP particles were optimized with sizes ranging from 25 to 40 µm, excellent adsorption efficiency in contrast to typical adsorbent like resin XAD and powdered activated carbon (PAC). Chen and coworkers proposed a multi‐template imprinting approach for up to sixteen polycylic aromatic hydrocarbons (PAHs) templates imprinted sol‐gel MIP adsorbent.^[^ ^139^ ^]^ The SPE adsorbent was demonstrated to possess high rebinding affinity, detection sensitivity, and satisfactory reusability for the extraction of multiple PAHs for seawater samples. Actually, the selectivity of a single template among compounds of multiple targets would be inevitably weakened in comparison with MIPs imprinted with a single template, because the recognition sites of each template decreased with the increase of remixing multiple binding sites, and thus the final recognition efficiency is integrally comprised of rebinding sites of multiple templates indeed.

### 3.4 Multi‐Functional Monomer Imprinting Technology

As we reviewed above, non‐covalent interactions between template molecules and functional monomers are relatively stable, contributing to rapid dissociating and rebinding during non‐covalent imprinting. These non‐covalent interactions like dipole‐dipole interactions, hydrogen‐bond formation, and electrostatic interactions can be enhanced by aggrandizing multiple interaction sites, which means introducing multiple functional monomers around template molecules. Afterwards, these multiple functional monomers provide different binding functional groups for complementary combination with different action regions or epitopes of template molecules (Figure 10B).^[^ ^140^ ^]^ Mosbach's group first demonstrated the possibility of synchronously imprinting with multiple distinct functional monomers for non‐covalent imprinting in 1993.^[^ ^141^ ^]^ They designed MIPs comprised of two functional monomers including MAA and 2‐vinlpyridine (2Vpy), which possessed carboxy and pyridinyl functionalities respectively. Such synthetic polymers were evaluated with better recognition capabilities as compared to MIPs with only a single MAA or 2Vpy, which confirmed the superiority of combining multiple functional monomers. Benefitting from the advantages of simultaneous targeting different moieties of template molecules, MIPs with more functionalities have been developed. Athikomrattanakul et al. fabricated MIPs with two complementary functional groups in parallel for thermometric sensor configuration of nitrofurantoin (NFT).^[^ ^142^ ^]^ The specific tailor‐made functional monomers complex consisted of first monomer diaminopyridine derivative (BMP) as the receptor for imide residue together with three kinds of (thio)urea‐based derivatives (1‐(4‐vinylphenyl)‐3‐(3,5‐bis(trifluoromethyl)phenyl thiourea, urea) (VTU, VFU) and second monomer 1‐(4‐vinylphenyl)‐3‐(pentafluorophenyl)thiourea (PTU)) to interact with nitro groups of NFT. Apart from some organic template molecules, multi‐functional monomer imprinting technology has also been applied for imprinting ions. For example, Li and coworkers reported novel Pb^2+^ ion imprinted polymers via synergistic interaction of dual functional monomers for solid‐phase extraction of Pb^2+^ in water samples.^[^ ^143^ ^]^ Two commonly used functional monomers like MAA and 4‐vinyl pyridine (4‐VP) were selected to form coordination complexes with Pb^2+^ ions. By virtue of the proton abstraction action of 4‐VP for proton acceptor MAA, Pb^2+^ ions could better bind with carboxyl groups, thus, these robust binding sites contributed to improving adsorption efficiency and selectivity. However, it is not so easy to discover reasonable multiple functional monomers which can interact with one template at different action sites. The synergetic binding sites should avoid mutual interference as far as possible and be able to keep stable under various polymerization reaction conditions or organic solutions. In addition, the explorations of new natural functional monomers or artificial monomer compounds also deserve more attention. And it remains a challenge for the further development of multi‐functional monomer imprinting technology to effectively utilize the collaborative rebinding sites of multifunctional monomers to enhance the final imprinting factor, binding affinity, recognition capability, and maximum rebinding efficiency.

### 3.5 Stimuli‐Responsive MITs

Stimuli‐responsiveness, as known as environmental responsiveness or intelligent responsiveness, refers to the capability of responding to several external stimuli with remarkable changes in properties like volume sizes (swelling or shrinking), topological shape, molecular chain structure, elasticity, solubility and structure colors, etc.^[^ ^144^, ^145^ ^]^ Commonly external stimuli include temperature, pH value, electric field, magnetic field, photonic irradiation, and so on. Generally, stimuli‐responsive MITs impart MIPs materials with these intriguing responsive properties, resulting in precisely controlled recognition and rebinding process of target template molecules. Although the external stimuli will inevitably lead to loss of memory of template imprints to some extent, the imprinting sites can still remain stable in the 3D structures of MIPs, which ensures the specific selectivity with a slight sacrifice of efficiency. Therefore, stimuli‐responsive MITs as new smart techniques have been widely applied for drug delivery system, separation science, sensor technology, and other biomedical fields.^[^ ^146^, ^147^ ^]^ In this section, we will review the mechanisms, advantages, disadvantages, and practical examples of several following stimuli‐responsive MITs.

#### 3.5.1 pH‐Responsive

**Figure 11:**
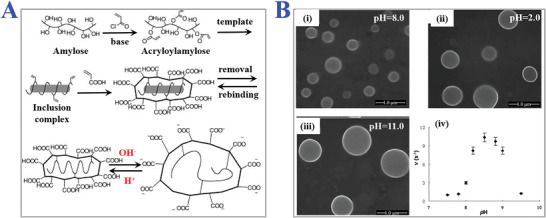
The applications of pH‐responsive MIPs. A) Synthesis scheme of amylose‐based MIPs and their pH‐responsive structural change. Helical inclusion‐complex formation between acryloyl‐amylose and monomers with ionizable units could be reversibly tuned depending on pH of solution. Reproduced with permission.^[^ ^149^ ^]^ Copyright 2003, Wiley‐VCH. B) SEM images of pH‐sensitive water‐soluble imprinted hydrogels under different pH value: i) 8.0, ii) 2.0, iii) and 11.0. iv) The catalytic efficiency of imprinted hydrogels in a buffer solution of different pH conditions. Reproduced with permission.^[^ ^152^ ^]^ Copyright 2010, Wiley‐VCH.

The pH‐responsive functionality of MIPs mainly relies on the pH‐sensitive functional groups, namely cationic groups (pyridine groups, amino groups, imidazole, dibuthylamine groups, methacrylatge groups, etc.) and anionic groups (carboxyl groups, etc.).^[^ ^148^ ^]^ When pH value of the external environment changed, these functional groups would undergo ionization and protonation process, destroying the cross‐linking degrees or stable interactions like hydrogen bonds between polymers’ skeletons, and eventually leading to the volume change of MIPs, as illustrated in **Figure** **11**.^[^ ^149^, ^150^, ^151^, ^152^ ^]^ Generally, under low pH condition, cationic groups tend to become protonated, and then the resultant increasing internal charge repulsions cause the swelling volume of MIPs (acid‐swelling). While under high pH conditions, with the decreasing of charge repulsions, cationic groups usually become less ionized, and other binding interactions within polymers themselves become tighter, finally causing the shrinking volume of MIPs (alkali‐shrinking). On the contrary, anionic groups will cause volume shrinking (acid‐shrinking) under low pH conditions, while causing volume swelling (alkali‐swelling) under high pH conditions.^[^ ^151^ ^]^ During the swelling or shrinking process, the spatial cavities of template imprints or the pore sizes of MIPs can be adjusted, making it possible to externally control the target rebinding and recognition process. Zhao and coworkers designed an enzyme mimic of horseradish peroxidase catalytic system, which combined pH‐responsive hydrogel with molecularly imprinted catalyst, as presented in Figure 11B.^[^ ^152^ ^]^ They fabricated tetrapolymer of NIPAM, 4‐VP, hemin, and acrylamide (AAm) to copolymerize with homovanillic acid (HVA) as template molecules based on precipitation polymerization. It was confirmed that this pH‐responsive MIP system possessed remarkable sensitivity under both acidic and alkaline conditions with obvious variations in volumes. The mechanism of swelling or shrinking was mainly attributed to the process of deprotonation and negative charge repulsion. When the repulsion overcame attractive forces, the MIPs could swell free under acidic conditions. This novel study indicated the feasibility of integrating stimuli‐sensitive monomers into MIP systems and explored the application of artificial enzymes or catalytic compounds.

Different from the component ratios during traditional MIPs procedures, the ratio of cross‐linkers to functional monomers is usually relatively lower to fabricate loose cross‐linked structures for better pH sensitivity, because highly cross‐linked structures of MIPs will hinder the slight changes caused by external pH conditions. Gong and his colleagues reported pH‐sensitive MIP nanospheres/hydrogel composites with controllable release capability of dexamethasone‐21 phosphate disodium (DXP) as a potential coating for better biocompatibility of implantable glucose biosensors.^[^ ^153^ ^]^ They set the molar ratio of functional monomers (2‐hydroxyethyl methacrylate (HEMA) and 2‐(diethylamino) ethyl methacrylate (DEAEMA)) to cross‐linkers (EGDMA) as 1:20. The final spatial structure was actually lowly cross‐linked, and the resultant MIPs showed higher loading capacity with pH‐sensitive release in the range of pH from 6.0–7.4, which endowed this new pH‐sensitive system with potentials for prolonging the lifetime of in vivo sensors. As mentioned above, a common synthesis route for fabricating pH‐responsive MIPs is to introduce pH‐sensitive monomers as functional monomers to interact with templates. Tao et al. proposed a novel molecular imprinting strategy for the preparation of pH‐responsive MIPs.^[^ ^149^ ^]^ They used amylose modified with acryloyl groups as host matrix, bisphenol A (BPA) as template molecule, and acrylic acid (AA) as functional monomers. Then the pH‐responsive MIPs were synthesized by typical radical polymerization in aqueous solution. They demonstrated that when the templates were copolymerized with functional monomers of ionizable units like carboxyl groups, the configurational structures of imprinted sites were reversibly changeable and well responsive to the pH value of the target solution. The resultant pH‐responsive MIPs system was expandable to other hydrophobic molecules as well as some metal ions due to the combination ability of abundant carboxylate groups.

#### 3.5.2 Thermo‐Responsive

**Figure 12:**
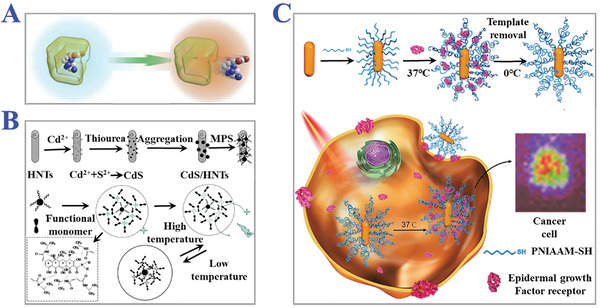
The applications of thermo‐responsive MIPs. A) The principle of thermo‐responsive release process of template molecules. Reproduced with permission.^[^ ^156^ ^]^ Copyright 2015, Elsevier Inc. B) Fabrication of CdS/halloysite nanotubes modified with thermal‐responsive MIPs layers as core‐shell photocatalysts for selective photo‐degradation of target tetracycline. Reproduced with permission.^[^ ^157^ ^]^ Copyright 2013, Royal Society of Chemistry. C) Thermal‐stimuli responsive MIPs nanoprobes for intracellular Raman imaging and visualization. The gold nanorods coated with MIPs layer could combine epidermal growth factor receptor (EGFR) under the temperature variation from 0 to 37 °C. Reproduced with permission.^[^ ^160^ ^]^ Copyright 2018, Wiley‐VCH.

Similar to pH‐responsive MIPs, the thermo‐responsive functionality is also based on the integration of thermo‐sensitive functional monomers during copolymerization processes. The thermo‐sensitive functional monomers are mainly comprised of amphipathic components like hydrophilic and hydrophobic groups. Such amphipathic components can change their spatial structures in response to the change in environmental temperature according to the principles of chemical thermodynamics and kinetics. Under different temperature conditions, these thermo‐sensitive monomers tend to form the most stable 3D conformation and reach kinetic equilibrium, which provides potential for controlled manipulation of combination between functional monomers and template molecules, such as easier removal of templates, enhanced mass transfer, and higher rebinding percentage. Low‐critical solution temperature (LCST) refers to the boundary temperature which causes the structural changes of thermo‐sensitive monomers.^[^ ^154^, ^155^ ^]^ At a temperature lower than LCST, hydrogen bonds between polymers’ skeletons act as the main intermolecular forces and determine the structural states of polymers. When the temperature was higher than LCST, these hydrogen bonds would be destroyed, and the increased hydrophobic interactions eventually caused the shrinkage of MIPs networks, as schemed in **Figure** **12**A.^[^ ^156^ ^]^ NIPAM, a commonly used thermo‐sensitive polymer, possesses an LCST at approximately 32 °C with a unique phase transformation capability depending on environmental temperature (Figure 12B).^[^ ^157^ ^]^ It could be anticipated that the weakened hydrogen bonds and increased hydrophobic interactions between the isopropyl groups of NIPAM could cause precipitation from aqueous solution at a temperature above LCST. Benefitting from the responsive abilities to thermal changes, NIPAM has been widely used as functional monomers for imprinting organic molecules, macromolecules, metal ions, biomolecules, etc. For instance, Zhao et al. proposed a thermo‐sensitive protein MIPs hydrogel for selective recognition of bovine serum albumin (BSA).^[^ ^158^ ^]^ They used N‐(3‐(Dimethylamino)propyl)‐methacrylamide (DMAPMA) as the main functional monomers to self‐assemble onto template proteins by electrostatic interaction, and NIPAM was chosen as an assistant functional monomer to impart polymers with thermo sensitivities. The volume of the resultant MIPs could be adjusted by thermo stimuli to realize the controllable recognition of target BSA proteins with higher affinity and specificity. Apart from introducing thermo‐sensitive monomers to bind with template molecules, there also exists another strategy to endow MIPs with thermo sensitivity, that is to modify typically fabricated MIPs with one thermo‐sensitive layer. Zhang and coworkers designed an efficient approach to obtain water‐compatible and thermo‐sensitive MIPs with facile surface‐grafting polymer brushes via RAFT polymerization.^[^ ^159^ ^]^ The NIPAM brushes were facilely grafted on MIP microspheres to improve dispersion stability in water and surface hydrophilicity. When the temperature exceeded 45 °C, the NIPAM brushes would collapse onto the MIP microspheres, decreasing the exposure of binding sites and hindering the rebinding efficiency, which realized the temperature gates for controlling target recognition. This route avoids adding other complicated functional monomers or polymerization methods, and enables molecular imprinting processes more flexible. Furthermore, such approaches would explore the applications of these intelligent thermo‐responsive MIPs for bioanalytical sensors, controllable drug delivery, molecular gating, and so on (Figure 12C).^[^ ^160^ ^]^

#### 3.5.3 Photo‐Responsive

**Figure 13:**
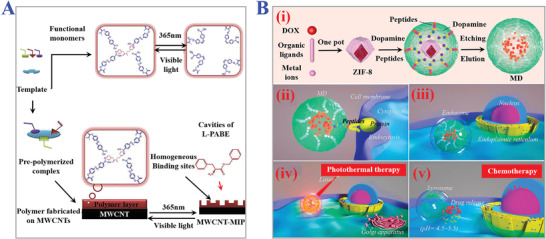
The applications of photo‐responsive MIPs. A) Synthesis process of multiwalled carbon nanotubes‐based MIPs for enantioselective recognition of L‐phenylalanine benzyl ester (L‐PABE) via integrating photo‐responsive functional monomer. Reproduced with permission.^[^ ^165^ ^]^ Copyright 2019, Elsevier Ltd. B) Preparation procedure of MIPs nanoparticles as photo‐thermal drug assays (i). The specific recognition and endocytosis process of capsule‐like MIPs nanoparticles for anti‐cancer therapy (ii–v). Reproduced with permission.^[^ ^166^ ^]^ Copyright 2021, Elsevier B.V.

Photonic irradiation is another conventional external stimulus including ultraviolet light, visible light, near‐infrared and infrared light, etc. Generally, under irradiation of such photonic stimulus, the isomerization of molecular chains or dissociation of functional groups would cause the changes in chemical or physical properties of polymers, which can be classified into four categories, including shift of hydrophilic‐hydrophobic balance, break of polymer junction, degradation of molecular chains and reversible cross‐linking.^[^ ^161^, ^162^, ^163^ ^]^ To prepare photo‐responsive MIPs, a feasible strategy is to introduce photo‐sensitive functional monomers which are comprised of three functional components involving photo‐sensitive segment, recognition segment, and polymerizable segment. Owing to their reversible photoisomerization features, azobenzene and its derivatives have been commonly applied in the synthesis of photo‐responsive MIPs. When exposed under ultraviolet light, the molecular conformation of azobenzene changed from stable trans‐isomer to cis‐isomer, with the reverse transformation to the original state occurring by irradiation of visible light. During the above processes, the recognition sites of MIPs tend to undergo changes in dipole moments and configuration geometry. Taking advantage of this, the controllable release of template molecules and uptake of targets can be achieved based on the reversible changes of internal imprints cavities (size, shapes, charges, chemical functionality) of MIPs through easy switches of ultraviolet light and visible light. Thus, integrating azobenzene and its derivatives into typically used functional monomers is a promising fabrication route for photo‐responsive MIPs (**Figure** **13**).^[^ ^164^, ^165^, ^166^ ^]^ Lam et al. designed water‐soluble 4‐[(4‐methacryloyloxy) phenylazo] benzenesulfonic acid (MAPASA) as azobenzene‐like functional monomers for the preparation of photo‐responsive MIP hydrogels to function in biocompatible aqueous media.^[^ ^167^ ^]^ Due to the reversible trans‐cis isomerization of MAPASA, such MIP hydrogels were demonstrated with high substrate affinity and the capacity to realize photo‐regulated release and uptake of paracetamol (N‐(4‐hydroxyphenyl) acetamide). The excellent properties imparted this photo‐responsive MIP hydrogel material with great potential for pharmaceutical separation and drug delivery systems.

Sol‐gel polymerization process has been considered an effective method for fabricating photo‐responsive MIPs. The resultant sol‐gel MIPs possess the advantages of thorough removal of template, stability under extreme conditions (strong acid, strong base, combustion, etc.), and the convenience of adjusting porosity and thickness.^[^ ^168^, ^169^ ^]^ These above advantages made photo‐responsive sol‐gel MIPs ideal candidates for more complicated applications. For example, Zhong and his coworkers developed organic‐inorganic MIP hybrid azobenzene materials through sol‐gel process for photoinduced recognition of 2,4‐dichlorophenoxyacetic acid.^[^ ^170^ ^]^ Similarly, they synthesized 4‐((4‐(3‐(trimethoxysilyl) propoxy) phenyl) diazenyl) phenyl 2‐(2,4‐dichlorophenoxy) acetate as azobenzene‐containing monomers according to Williamson etherification. The sol‐gel procedure contributed to building the silicate frameworks and unoccupied cavities of MIPs with organic moieties integrated. The resultant photo‐responsive MIPs were endowed with photo‐switchable and selectivity of target molecules upon irradiation at 360 nm and 440 nm, which could be applied for smart chemical sensors. However, the practical applications of photo‐responsive MIPs still faced challenges. The relatively slow transformation time of trans‐cis isomerization of photo‐sensitive functional monomers restricted the development of MIPs for rapid analysis and dynamic sensing. Besides, the irradiation conditions like limited wavelength and intensity could also cause inevitable and metamorphic damages to some targets or substrates. Therefore, more research emphasis should be put on synthesizing suitable photo‐sensitive functional monomers for MIPs to meet the flexible acquirements of diverse applications.

#### 3.5.4 Magnetic‐Responsive

**Figure 14:**
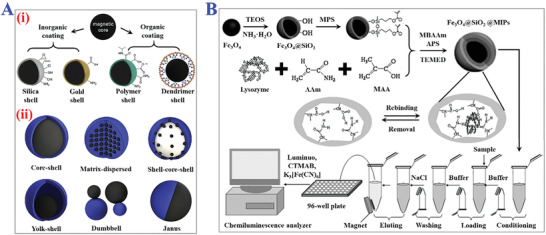
The applications of magnetic‐responsive MIPs. A) (i) MIPs comprising of magnetic core and organic or inorganic shells. (ii) Different constructions of magnetic MIPs. Black part is the magnetic component and blue part is MIPs part. Reproduced with permission.^[^ ^171^ ^]^ Copyright 2019, Elsevier B.V. B) Scheme of the lysozyme detection system based on Fe_3_O_4_@SiO_2_@MIPs. The magnetic responsiveness could realize easy separation of target lysozyme for sensitive CL. Reproduced with permission.^[^ ^172^ ^]^ Copyright 2010, Elsevier B.V.

A typical strategy for the fabrication of magnetic‐responsive MIPs is to adopt magnetic materials as supporting substrates or functional components into polymers (**Figure** **14**A).^[^ ^171^ ^]^ Benefitting from the excellent magnetic responsiveness and easy operation of performing magnetic control, these magnetic components can be directionally removed from the MIPs upon external magnetic or electric fields. Such advantage provides feasible routes for efficient removal of template molecules during the fabrication step and effective purification of resultant polymers from compounds or medium solution, which avoids complex centrifugation or filtration processes and ensures the purity of polymers with stable recognition specificity. Hence, magnetic‐responsive MIPs have been widely applied in sample preparation, magnetic‐basic solid‐phase separation, controllable drug delivery, enzyme immobilization, cell sorting, and so on (Figure 14B).^[^ ^172^ ^]^ Mosbach first proposed the concept of magnetic MIP beads for drug radioligand binding assay in 1998.^[^ ^173^ ^]^ The superparamagnetic molecularly imprinted copolymer beads comprising of MAA and 1,1,1‐trimethylolpropane trimethacrylate (TRIM) were confirmed with capacity of easy separation from solution by the inclusion of magnetic iron oxide and high binding affinity to target (S)‐propranolol. With the development of magnetic materials for MIPs, Fe_3_O_4_ nanoparticles have been considered as a commonly used affording substrate, which is conventionally synthesized by coprecipitation method or solvothermal reduction method, followed by the modification with functional groups on their surfaces. Then several polymerization methods like surface imprinting, precipitation imprinting, or grafting methods are widely chosen to prepare magnetic‐responsive MIPs with spherical shape, particle, or core‐shell structure. For instance, Mei and his coworkers designed magnetic molecularly imprinted Fe_3_O_4_@SiO_2_ nanoparticles for specific recognition of lysozyme.^[^ ^172^ ^]^ They transformed the surface of Fe_3_O_4_ nanoparticles to silica shells with tetraethyl orthosilicate (TEOS) by sol‐gel process, and then the *γ*‐methacryloxypropyltrimethoxysilane (MPS) was used to attach the surface with abundant double bond for growth of MIP film. The modification of functional groups aimed at enhancing the following covalent attachment of specific ligands including template lysozyme, AAm, and MAA. It was demonstrated that sufficient magnetic components were encapsulated in the resultant MIPs for an easy magnetic separation process. Such magnetic‐responsive Fe_3_O_4_@SiO_2_ MIPs showed excellent template recognition properties and high adsorption capacity. And all the characterization indicated that these multifunctional magnetic materials would explore the potential applications in bio‐separation, cell labeling, or bioimaging fields.

Suspension polymerization is another simple and suitable method for the preparation of magnetic‐responsive MIPs, which avoids disadvantages like excessive remnants, low extraction capacity, and laborious surface modification compared with other polymerization methods. Li et al. developed a novel microwave heating method for the preparation of magnetic‐responsive MIP beads for trace triazines analysis in complicated samples.^[^ ^174^ ^]^ They used polyethylene glycol (PEG) modified Fe_3_O_4_ as magnetic cores, and then template atrazine and monomer MAA were self‐assembled by microwave heating during suspension polymerization. The porous magnetic beads with spherical shape were characterized by a narrow diameter distribution (80–250 µm), magnetic property (M_s_ = 0.491 emu g^−1^), and thermal stability under 260 °C. Apart from Fe_3_O_4_ and its derivatives, some other magnetic substrates like azide‐functionalized nanoparticles, titanium dioxide (TiO_2_), and QDs have also been investigated for further development of magnetic‐responsive MIPs‐based sensors or artificial antibody synthetic fields.

### 3.6 Other Novel Technologies

Above several MITs are widely used techniques in typical preparation processes of MIPs. These methods have been applied to fabricate diverse molecular imprinted materials in scientific fields of practical chemical and biomedical engineering. With the development of characterization methods and synthesis strategies, some other novel MITs have also been proposed to meet the requirements of some particular applications.

#### 3.6.1 Controlled/Living Free Radical Polymerization (CLFRP)

**Figure 15:**
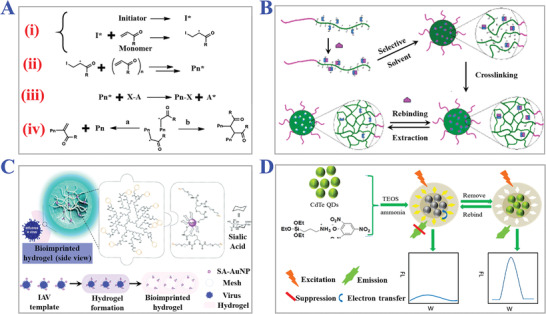
The applications of other novel MITs. A) The mechanism of reversible addition‐fragmentation chain transfer polymerization (RAFT). i) Initiation. ii) Propagation. iii) Chain transfer. iv) Termination by disproportionation and combination. Reproduced with permission.^[^ ^178^ ^]^ Copyright 2016, Elsevier Ltd. B) Synthesis procedure of MIPs nanospheres via self‐assembly of deblock copolymers. Reproduced with permission.^[^ ^183^ ^]^ Copyright 2006, American Chemical Society. C) Construction of bio‐imprinted (influenza A virus) MIPs hydrogel with dual responsiveness including volume (swelling and shrinking) and color changing (purple to pink). Reproduced with permission.^[^ ^185^ ^]^ Copyright 2020, Royal Society of Chemistry. D) Schematic illustration of the dummy MIPs‐capped CdTe QDs for fluorescent sensing of TNT. Reproduced with permission.^[^ ^192^ ^]^ Copyright 2013, American Chemical Society.

To obtain MIPs with well‐defined molecular weight, topological structure, low dispersity, precise composition, and exact functionality, controlled/living free radical polymerization (CLFRP) methods have been thoroughly investigated, including RAFT, ATRP, nitroxide‐mediated polymerization (NMP), initiating transfer terminator method and nitrogen oxides‐controlled stable free radical polymerization (SFRP).^[^ ^175^, ^176^, ^177^ ^]^ The growth processes of polymer molecular chains during polymerization tend to be slower via thermodynamic control. Thus, the rates of chain growth and chain relaxation can reach a better balance. The resultant network of MIPs will be more homogeneous within a narrow range of molecular chain length. Generally, free radical polymerization involves a chain transfer reaction and a chain termination reaction. The critical feature of CLFRP is to reduce the concentration of active free radicals and the rate of chain termination. RAFT is a typical CLFRP method due to its tolerance for amounts of functional monomers and flexibility for diverse designs. During RAFT procedure, special agents with high chain transfer constant like double sulfur ester and its ramifications are usually used to enhance degradation transfer of free radical, as shown in **Figure** **15**A.^[^ ^178^, ^179^ ^]^ Similarly, ATRP procedure, another new class of CLFRP, tends to use transition metal catalysts as halogen atom transfer assays for mediating the propagation and constructing reversible “promoting‐inactivation” reaction to minimize chain termination rates under relatively mild reaction conditions. Yang and his coworkers developed *β*‐estradiol imprinted nanotubes within a porous anodic alumina oxide (AAO) membranes for the chemical separation of target molecules by surface‐initiated ATRP route.^[^ ^180^ ^]^ They grafted 2‐bromo‐2‐methylpropionyl bromide as ATRP initiator on the silanized AAO membranes to induce the following copolymerization of *β*‐estradiol, 4‐VP, and EGDMA. CuBr and 1,4,8,11‐tetraazacyclotetradecane (Me_4_Cyclam) acted as halogen atom transfer assays to control the growth of polymer chains onto the nanotubes. The final MIPs were tailored with uniform pores and adjustable thickness, and the binding affinity to targets was confirmed 11‐fold higher than common bulk MIPs. However, there still exist some challenges limiting the further development of CLFRP, such as realizing polymerization within the water or other liquid medium, complete elution of transition metal catalysts from MIPs, complex synthesis of initiators or iniferter, and so on. Therefore, researchers should pay more attention to the above problems to explore wider applications of CLFRP.

#### 3.6.2 Self‐Assembly of Block Copolymer

To prepare MIPs with core‐shell structures, self‐assembly of block copolymers has been adopted as a superior method in recent years.^[^ ^181^, ^182^ ^]^ Block copolymers tend to remain in micelles states based on the different solubility among selective solvents. Insoluble blocks can act as solid core or supporting substrates for anchoring soluble blocks which act as compositions of shell layer. During the typical self‐assembly procedure of MIPs block copolymers, first, the block copolymers with sectional block, which loaded combining sites to corresponding templates, were synthesized. Then the block copolymers combined templates based on interactions like hydrogen‐bonding, covalent interactions, etc. After transferring to selective solvents, the block copolymers would self‐assemble towards spherical core micelles with soluble block forming shell micelles. Subsequently, the micelles with core‐shell structure gradually became highly cross‐linked during polymerization processes. Finally, the template molecules were removed in such polymerized core‐shell micelles to construct desired MIPs. Compared with the methods which establish core‐shell MIPs by integrating with supporting substrates like Fe_3_O_4_ or QDs, the overriding advantage of this self‐assembly strategy is to avoid the modification or pretreatment process of core materials and reduce the complexity of interactions between core, shell, and template materials. The different block sequences within one block copolymer make up the functional components of final MIPs synergistically (Figure 15B).^[^ ^183^ ^]^ Wooley et al. proposed a method to realize sequence control of triblock copolymers with unique toroidal morphology.^[^ ^184^ ^]^ They synthesized poly[(tert‐butylmethacrylate)‐block‐(2‐hydroxylethylmethacrylate)], which was modified with 2‐acrylamide‐6‐carboxylbutyl amidopyridine and methacryloyl side group as cross‐linkable functional groups. 1‐alkyluracil and 1‐alkylthymine derivatives were selected as templates to form complexes with block copolymers by hydrogen bonding. Then the precursor complexes were self‐assembled in cyclohexane to form core‐shell nanostructures. The resultant MIPs exhibited high rebinding affinity to targets with better dispersibility due to the self‐assembly process of micelles. Furthermore, attributed to the self‐assembly of block copolymers approach, the performance of MIPs could be further adjusted by changing the block length, sequence arrangement, and group polarity.

#### 3.6.3 Dual/Multi Responsive MIPs

In Section 3.5, we have reviewed several single responsive MIPs which respond to pH, thermo, photo, and magnetic stimuli. Based on these intelligent single responsive MIPs, dual/multi responsive MIPs have been proposed by combining two or more external stimuli responsive materials into synthesis processes of MIPs, as presented in Figure 15C.^[^ ^185^ ^]^ For instance, Yan et al. designed temperature‐magnetic responsive MIP nanotubes imprinted with 2,4,5‐trichlorophenol by surface imprinting technique.^[^ ^186^ ^]^ NIPAM was used as thermoresponsive monomers to be copolymerized with functional monomers MAA onto the surface of Fe_3_O_4_ nanoparticles formed through dehydration. Taking advantage of the dual‐responsive MIPs, they successfully realized circular and complete separation of target molecules and MIPs from solution containing complex compounds. At 60 °C, MIPs could recognize and bind with target at a fast rate. The external magnetic field was conducted to purify the target‐MIPs hybrid due to the excellent magnetic responsiveness of Fe_3_O_4_ components. As the temperature decreased to 20 °C, the thermo responsiveness of NIPAM could inflate the polymer networks and release the targets. Finally, the target and the MIPs would be separated by magnetic stimuli again. Compared to dual responsive MIPs, the preparation of multi‐responsive MIPs are more difficult due to the difference in properties among multi‐responsive monomers or substrate materials. Therefore, only a limited number of articles reported multi‐responsive MIPs. Zhang and his colleagues described a facile and highly efficient approach to fabricating monodispersed MIP microspheres with multiple responsive (thermo, pH, photonic) template binding capacities through surface‐initiated RAFT polymerization.^[^ ^187^ ^]^ First, the azo functional monomers containing MIP layer were grafted onto the core polymer beads. Then NIPAM and 2‐(dimethylamino)ethyl methacrylate (DMAEMA) were copolymerized as brushes onto the preformed photonic responsive microspheres. The resultant multi‐responsive MIPs showed respective responsiveness to light, temperature, and pH stimuli. The specific binding affinity and binding‐release circulations were proved efficient, which indicated the excellent properties of such multi‐responsive MIPs. This strategy provided a promising way for the synthesis of multi‐responsive MIPs with less‐complicated optimization and more intelligent functionalities, implying the feasibility of simultaneously combining MITs with various stimuli‐responsive materials.

#### 3.6.4 Dummy Imprinting Method

In general, suitable template molecules usually play a critical role during the typical fabrication procedure of MIPs. According to the properties and functional units of template molecules, corresponding types of binding interactions and polymerization methods should be chosen to provide the best reaction environment. However, in some cases, the control of the target template molecules is difficult. Specifically, some template molecules are expensive to purchase or need complex chemical synthesis processes, while some original templates may not remain stable in the solvent or polymerization process, which would inevitably influence the imprinting efficiency and functionality of resultant MIPs.^[^ ^188^, ^189^ ^]^ In order to overcome the difficulty of finding template molecules for MIPs, a novel strategy named as “dummy imprinting method” was developed, which uses structurally analogous compounds with similar functional sites for specific recognition to replace the original template molecules.^[^ ^190^ ^]^ Such a strategy was first proposed by Takeuchi et al. in 2000.^[^ ^191^ ^]^ Chen and his coworkers designed dummy MIPs‐capped CdTe QDs for fluorescent sensing of TNT, as illustrated in Figure 15D.^[^ ^192^ ^]^ They chose trinitrophenol (TNP) as a dummy template to replace TNT, since TNP had a similar shape and functional groups with more stability and safety compared to TNT. The TNP‐imprinted MIP@QDs were demonstrated to retain high selectivity and sensing capacity to target TNT. More importantly, the feasibility of dummy imprinting strategy was proved and such MIP@QDs‐based systems were considered as ideal candidates for constructing intelligent fluorescent sensors. Although dummy imprinting method has been increasingly applied to satisfy the diverse complicated needs of preparing MIPs in recent years, there still exist some problems to solve. After dummy templates act as alternative templates to replace the original molecules, how to differentiate the recognition capacity of MIPs to original templates and dummy templates is a tough trouble. Additionally, the interactions between dummy templates and other reagents during polymerization processes could influence the properties of MIPs such as solubility, hydrophilicity, hydrophobicity, and some stimuli responsiveness. When these MIPs were used for specific or steerable recognition of original templates, the above changes of properties would hinder the rebinding affinity and lower the final efficiency. These problems would inevitably impede the wider applications of dummy imprinting methods for the development of MIPs.

## 4 Biomedical Applications

With the further development of polymer materials, polymerization strategies, and controllable reaction microenvironment in recent years, MIPs have been integrated into many practical applications, mainly including sample separation sciences, sensing techniques, efficient catalysis, drug delivery, and so on.^[^ ^193^, ^194^, ^195^ ^]^ The excellent flexibility of MIPs enabled the customization of desired properties to satisfy the requirements as far as possible by selectively adjusting the raw segments and imprinting procedures. Due to the superior capacity of affinity recognition and relatively convenient operation processes, MIPs showed promising application potentials in these fields. Meanwhile, there still existed some challenges for MIPs in the exploratory phase of some particular fields, which hindered the wider evolution. In this section, some exciting and universal applications of MIPs will be reviewed.

### 4.1 MIPs for Separation Techniques

Separation techniques have been mostly used for sample pretreatment including preconcentration and purification, which prepared the sample with optimal status for subsequent trace/ultratrace level analysis. The appropriate separation technique is very important to promote treatment efficiency and sample quality. For this reason, several sample extraction methods have been proposed like chromatographic separation, SPE, and solid phase microextraction (SPME) to extract pure compound or tailor‐made components with desired selectivity from initial samples. However, some drawbacks like large usage of organic solvent and cross‐pollution of sorbents medium inevitably led to low enrichment efficiency and purity. MIPs, as alternative sorbent materials with excellent selectivity, high affinity, and low risk of cross‐pollution, have been considered to be integrated into separation techniques and shed light on the outstanding performances of improving extraction efficiency of targets.

#### 4.1.1 Chromatographic Separation

**Figure 16:**
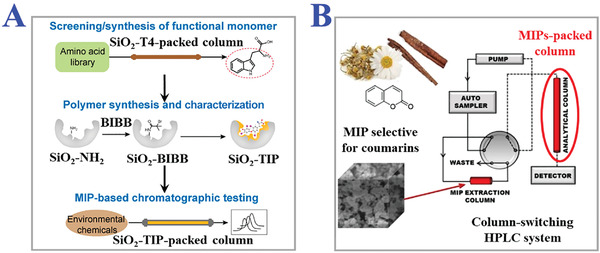
The applications of MIPs for chromatographic separation. A) Schematic workflow of the MIPs‐packed column‐based chromatographic testing. Reproduced with permission.^[^ ^199^ ^]^ Copyright 2020, American Chemical Society. B) Scheme of on‐line HPLC system via packing selective MIPs into the column cartridge, equipped with a column switching valve for transferring pre‐concentrated coumarins into MIPs‐packed column. Reproduced with permission.^[^ ^200^ ^]^ Copyright 2017, Elsevier B.V.

Recently, commonly used chromatographic separation techniques mainly contain capillary electrochromatography (CEC), gel permeation chromatography (GPC), gas chromatography (GC), HPLC, etc.^[^ ^196^, ^197^, ^198^ ^]^ In a typical chromatographic separation procedure, the mobile phase was driven through the stationary phase. During this process, different components of samples in mobile phase would be separated and distributed in different areas of the stationary phase due to the differences in physicochemical properties like solubility, polarity, affinity, adsorption capacity, and so on. The stationary phase played the role of sorbent materials to provide adsorption superficial area, flow resistance, and specific affinity to the target components. Such specific affinity and recognition capacity can be also realized by the mechanism of MIPs, and therefore MIPs could act as stationary phase materials for chromatographic separation. Tailor‐made MIPs materials could be packed in a chromatographic column for subsequent extraction analytes, as shown in **Figure** **16**A,B.^[^ ^199^, ^200^ ^]^ When the samples loaded in mobile phase flowed through the chromatographic column with high pressure, the MIPs materials with functional sites for specific recognition of target templates could fully react with the samples at the solid‐liquid interface. The target components would fill the void space of molecular imprints, and after multiple elution operation, the desired components could be extracted from the MIPs‐targets compound in the chromatographic column.

For instance, Xu et al. designed a coupled LC‐MS (liquid chromatography‐mass spectrometry) system to realize affinitive separation and online identification of anti‐tumor components by packing target analog imprinted polymer in a chromatographic column.^[^ ^201^ ^]^ They synthesized three templates‐imprinted MIPs, where functional monomer MAA, cross‐linker EGDMA, initiator AIBN, and several porogens were copolymerized with templates harman, harmaline analog, and harmine by a typical bulk polymerization procedure. Such bulk MIPs were dry‐packed into stainless steel columns for subsequent chromatography study. Compared with the nonimprinted blank polymers, the targets‐imprinted MIPs in column showed higher affinity and selectivity to anti‐tumor compounds harmine and harmaline after MIP‐LC‐MS separation. Meanwhile, they changed the ratio of porogens during MIPs preparation. It was demonstrated that the mass ratio of porogens actually influenced the target's binding efficiency and extraction performance. Suitable ratio of porogens could maximize the functional sites and affinity while ensuring the least targets escape proportion. This work indicated the potential of integrating MIPs materials with chromatographic separation for molecular screening and herbs extraction with better accuracy and efficiency. In addition, MIPs‐packed chromatographic column could improve the extraction speed and realize fast chromatography. Fast chromatography was developed for large‐scale, high throughput, convenient purification or extraction of samples, so the MIPs bearing easy fabrication procedure and low cost could satisfy the economic needs of fast chromatography. Meng and his coworkers fabricated shikimic‐imprinted MIPs, and packed the polymer particles as flash chromatography columns for extraction of shikimic acid from Chinese star anise.^[^ ^202^ ^]^ Such MIPs column reached adsorption equilibration with a low time course and could be reused for 4 cycles. Hence, the MIPs column could be applied for fast chromatography extraction of targets from complex and vast samples with the advantages of rapid speed, reusability, high efficiency, and low cost.

Apart from MIPs‐packed chromatography column, monolithic column is used as another stationary phase. Compared with MIP‐packed column, monolithic column is often directly synthesized in situ within stainless columns or capillary columns, which avoids the additional washing and packing processes, and bears easier preparation, lower backpressure, and faster mass transport efficiency.^[^ ^203^ ^]^ Chen and his colleagues designed monolithic MIPs column for HPLC semi‐preparative separation by combining in situ synthesis and surface‐initiated imprinting.^[^ ^204^ ^]^ They first packed the silica beads and initiator into empty stainless steel HPLC column, and then 4‐4’‐azobis(4‐cyanovaleric acid) (ACVA) was imprinted on the surface of the activated silica bead. The resultant monolithic MIPs column exhibited excellent retention capacity for template emodin from plant extract at a semi‐preparative scale. This method just consumed a small amount of template for the fabrication of monolithic column, which provided a new approach for imprinting some natural but difficult to obtain products. During the preparation of monolithic MIPs columns, organic polymers have been frequently used to endow the monolithic column with the ability to remain stable under solvent environments of different pH values, temperature, and polarity. However, when monolithic MIPs columns were immersed in some organic mobile phases, the inevitable shrinking or swelling destroyed the structure of molecular imprints and influence the binding efficiency. To solve this problem, an organic‐inorganic hybrid technique combining organic polymer with silica gel process was developed. Wang et al. proposed capillary monolithic MIPs column by integrating multi‐template imprinting with organic‐inorganic hybrid strategy.^[^ ^205^ ^]^ Four isomers of ractopamine were imprinted into an activated fused‐silica capillary with different ratios of methanol and toluene as a polymerization solvent. The nature of the amino and Si—OH on the surface of the prepared organic‐inorganic hybrid capillary column could change with the pH value changing, so the column could keep high selectivity for templates under buffer conditions of different pH values.

As reviewed above, both MIPs‐packed columns and monolithic MIPs columns have been widely used as stationary phase for chromatographic separation techniques. The two forms could simplify the conventional steps and accelerate the analysis speed of chromatographic separation, which enabled the MIPs‐based chromatographic for wider applications in the field of environmental monitoring, food safety, biomedical analysis, and drug extraction.

#### 4.1.2 Solid Phase Extraction (SPE)

SPE, another important separation technology, has been developed to fulfill increasingly manifold requirements of sample pretreatment in recent years. Compared with traditional chromatographic separation techniques, SPE showed more superiority in inexpensive time cost, convenient operations, and lower ambient pressure for sample uploading and elution. In common SPE procedures, solid absorbents acting as stationary phases have been used to realize the extraction of targets based on the heterogeneity of adsorption affinity between different components in the sample medium. These solid absorbents should satisfy the following demands such as designable pore size distribution, amorphous structures, compact holistic configuration, etc., while these properties are exactly what MIPs materials do well. Benefitting from the artificially tailor‐made recognition sites, MIPs could easily realize sensitive capture of targets and simple dissociation which separately corresponded to processes of purification and collection. Therefore, MIPs for SPE, also namely MISPE, have been widely applied for target compounds extraction of chem‐biological samples, environmental pollutants, drug screening, food safety inspection, and so on.^[^ ^206^, ^207^, ^208^ ^]^

**Figure 17:**
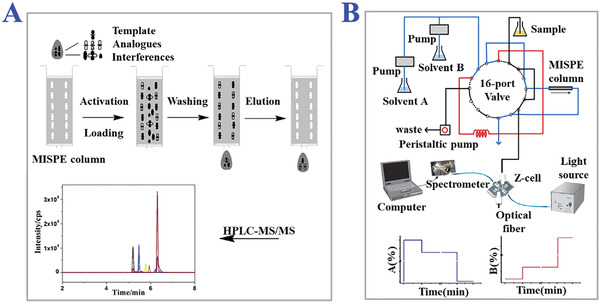
The applications of MIPs for solid phase extraction (MISPE). A) Scheme of off‐line MISPE method coupled with HPLC‐MS/MS for subsequent determination of seven nitroimidazoles. Reproduced with permission.^[^ ^209^ ^]^ Copyright 2017, Elsevier B.V. B) Schematic illustration of on‐line MISPE system equipped with fiber‐optic spectrophotometry for simultaneous detection of 4‐nitrophenol. Reproduced with permission.^[^ ^212^ ^]^ Copyright 2014, Wiley‐VCH.

Generally speaking, a typical MISPE procedure consists of four main steps: synthesis of suitable MIPs sorbent, package of SPE column and sample uploading, washing away interferents, and elution of target components. Considering the needs of desired shapes, binding sites, porosity, permeability, adsorption‐specific surface area, and tolerance in organic solvent, diverse MITs have been adopted to fabricate tailor‐made MIPs sorbents. So far, mostly used MISPE often operates as an off‐line mode, where operation staffs need to manually load reagents including sample, sorbent, solvent, and additives, and then collect the purified sample component (**Figure** **17**A).^[^ ^209^ ^]^ For example, Su et al. developed mixed templates‐imprinted bulk MIPs as sorbents of the off‐line MISPE procedure for effective recognition of seven kinds of targets in fish farming water samples.^[^ ^210^ ^]^ They chose any two pairs of items among sulfonamide (SA) and its six other ramifications as mixed templates. The solid sorbents were fixed with two (polytetrafluoroethylene) PTFE frits and padded uniformly in a MISPE tube for multi‐selectively adsorption of corresponding template groups, and then filtered residue was obtained after elution and drying. The analysis results revealed that such MISPE tube possessed a high sample elution ratio and low relative standard deviation. It was worth noting that more convenient extraction operation could be achieved by taking advantage of the magnetic responsiveness from MIPs sorbents under the switch of external magnetic field. Shi and his coworkers combined surface MIT and dummy imprinting strategy to prepare rhapontigenin (as dummy template of target resveratrol)‐imprinted MIPs based on the superparamagnetic core‐shell particle support.^[^ ^211^ ^]^ They confirmed that such magnetic MIPs sorbents showed high adsorption capacity and efficient extraction equilibrium when packaged in MISPE procedure. Different from typical steps of encapsulation SPE tube, such magnetic MIPs sorbents could be easily collected by an external magnetic field for subsequent elution and detection, which reduced the residual loss and improved extraction reliability at the same time.

Another operating pattern is on‐line mode, which combined MISPE as an upstream purification procedure with other downstream detection techniques like liquid chromatography (LC) or GC for subsequent automatic analysis of targets. By coupling such two parts into an integral and continuous analytical chain, sample contamination and solvent usage could be reduced to a certain extent. There exist two main construction routes of on‐line MISPE systems. One is to connect extraction columns with detection columns that are packed alone, and the other is to assemble monolithic columns, as shown in Figure 17B.^[^ ^212^ ^]^ For instance, Wang's group developed a chrysoidine‐imprinted MIP silica gel microsphere based on the surface molecular imprinting method integrated with sol‐gel process.^[^ ^213^ ^]^ They encapsulated the above MIPs sorbents into a cylindrically shaped microcolumn as MISPE packing unit, which was then combined with an HPLC detection unit to form an on‐line MIP‐SPE‐HPLC system. A six‐port injector valve was designed to realize the switch of the system function from the “load” to “inject”, where “load” position only activated the SPE unit to extract samples and “inject” position transferred such purified sample solutions into HPLC unit by HPLC mobile phase for analysis. This novel system could repeat the above extraction and analysis processes just by turning the injector valve, which imparted it with excellent reproducibility. However, such analogous on‐line MISPE system with separated packed functional columns still suffered from the dilemma including the controllable injection of multiple necessary solvents, postprocessing of the extracted residues like drying and redissolution, compatibility of different solvents and so on. Hence, assembling monolithic columns showed more advantages in solving the above problems during operations of practical on‐line MISPE systems. Lee et al. proposed a novel in‐column MISPE concentrator coupling with CE for urine sample analysis.^[^ ^214^ ^]^ They adopted light‐emitting diode‐induced polymerization method to coat the porous MIP sorbent layer inside the separation capillary via on‐line synthesis. In this configuration, the analytes bound in the MIPs sorbents could undergo desorption in situ as a determinant for subsequent CE analysis. Such a monolithic on‐line MISPE system successfully improved the separation and detection efficiency due to the compact construction, rapid adsorption equilibrium and dissociation, and small reagent consumption. What's more, the reproducibility of measurements and sorbent stability were also proved to be excellent.

Certainly, by means of the superior specific recognition property of MIPs, some other forms of SPE could also be designed with MIPs materials, such as in‐line SPE, dispersive SPE, matrix solid‐phase dispersion, and so on. In brief, the development directions of SPE have been broadened via the integration of MIPs. Abundant MITs and extensive polymer materials actually endowed SPE with infinite possibilities for wider applications.

#### 4.1.3 Solid Phase Microextraction (SPME)

As a promising application and development direction of classical SPE, SPME techniques, chasing for miniaturization and high‐throughput, have successfully lowered separation time and the usage of excessive solvent, and improved the final extraction efficiency. In recent years, SPMEs have been developing mainly into two major categories: fiber‐based SPME technique and in‐tube SPME technique. Similarly, MIPs materials could participate in the construction of SPME, known as MISPME. Instead of some rare or commercially unavailable polymeric materials with unadjustable physicochemical properties, MIPs with tailored functions could expand the options of raw materials and satisfy the complex demands for more applications.

**Figure 18:**
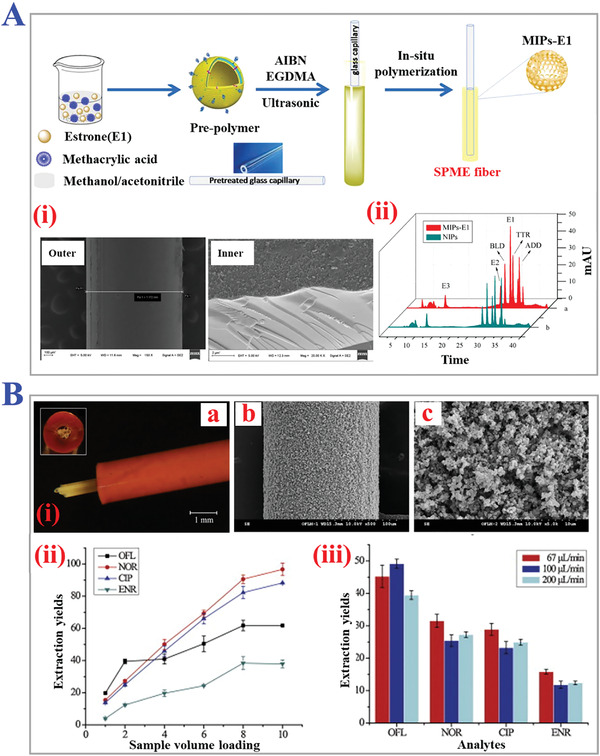
The applications of MIPs for solid phase microextraction (MISPME). A) Schematic illustration of MIPs‐estrone‐coated SPME fiber for EDCs analysis via in situ polymerization on inner and outer surface of the capillary. i) SEM images of outer surface and inner surface of MIPs‐E1‐SPME fibers. ii) Chromatogram of selectivity study for six EDCs of MIPs‐coating and blank groups. Reproduced with permission.^[^ ^216^ ^]^ Copyright 2018, Elsevier B.V. B) (i) Micrograph of the multiple fibers packed tube (a). SEM images of the ofloxacin imprinted SPME fiber (b,c). Effect of sample volume loading (ii) and analytes loading rate (iii) on the extraction yields of multiple MIP‐fibers packed PEEK tubes. Reproduced with permission.^[^ ^219^ ^]^ Copyright 2012, Elsevier B.V.

In fiber‐based SPME procedure, polymeric stationary phase coated on a fiber bears the extraction functions, where the adsorption occurs on the external surface of the fiber. Numerous materials have been chosen as basal fibers like glass capillary, fused‐silica, stainless steel, sol‐gel porous silica, and so on. MIPs could be easily coated on these SPME fibers with the help of several in situ polymerization methods, directly triggering the polymerization of MIP reagents on the surface of fibers. Hu and his colleagues designed a silicon MISPME fiber with ultra‐thin Sudan I‐imprinted coating layer via surface RAFT.^[^ ^215^ ^]^ The MIP coating layer was measured with just 0.55 µm thickness, and its dense, homogeneous, and porous microstructure provided sufficient binding sites for selective elution and desorption of analytes. They coupled such MISPME fibers with subsequent LC and mass spectrometry (MS) combination detection. Much lower limit of detection (LOD) and higher sensitivity were realized for monitoring of target trace Sudan dyes. Wang's group established novel estrone (E1) MIPs‐coated capillary for endocrine disrupting chemicals (EDCs) analysis based on SPME/HPLC, as presented in **Figure** **18**A.^[^ ^216^ ^]^ The several targets and functional monomers were in situ polymerized on the inner and outer surface of the capillary. Such MIPs‐coated capillary successfully realized the enhancement of enrichment and extraction ability to six target EDCs, which were measured double of commercial fibers. Apart from simply coating MIPs layer on the surface of basal fibers, some approaches utilizing composite constructions like parallel combination, nested structure, etc., have also been developed. For example, Tan et al. proposed BPA‐imprinted MISPME fiber by replicating the sleeve structure with controllable thickness on fused‐silica capillaries.^[^ ^217^ ^]^ They inserted a capillary into another larger bore capillary to form a mold, and then filled the interspace between two capillaries with MIP reagents under UV photo‐irradiation polymerization. Outer capillary was etched by hydrofluoric acid to reveal the MIPs coating layer. Such a simple approach could fabricate MISPME fibers with precise coating thicknesses via tuning the diameters of capillaries.

Combining the principle of capillary microextraction and fiber‐based MISPME, in‐tube MISPME technique was proposed with better mechanical stability, lower backpressure, higher elution sufficiency, and lower time consumption. In‐tube MISPME adopted capillary columns like an open tubular capillary, packed capillary, monolithic capillary, etc. with suitable stationary MIPs sorbent coated on inner surface for sample extraction. During in‐tube MISPME procedure, target analytes could be extracted and desorbed more efficiently via the higher capacity of capillary, and the switch of solvent stream could be supported by capillary switching techniques.^[^ ^218^ ^]^ Li and his coworkers established longitudinally packed MIP‐fibers into polyetheretherketone (PEEK) tube as a fiber‐in‐tube MISPME unit for sensitive analysis of antibiotic drugs (Figure 18B).^[^ ^219^ ^]^ Multiple MIPs coated fibers were manually inserted into hollow PEEK tube. Such design actually enhanced the extraction capacity by utilizing the advantages of fluid dynamics of longitudinal channels. In addition, in‐tube MISPME coupled with HPLC, CE, or GC process could serve as an automation system comprising of extraction, desorption, and detection. Micro automated samples bring shorter analysis time and nearly no environmental pollution, which are valuable in such automation systems as commercial columns for practical applications. However, there still exist some drawbacks of the above in‐tube MIPSPME. For example, capillary and tube would be easily blocked due to the viscidity or amphipathy of samples, and the miniaturization of flowing lines should be more integrated while guaranteeing efficiency, and so on.

### 4.2 MIPs for Chemical and Biological Sensing

In recent years, the development of sensing techniques in the field of modern analytic science has attracted more and more interest due to the increasing demands for precise and sensitive characterization of target analytes. Outstanding selectivity and sensitivity are necessary for the construction of most chemical or biosensors. As introduced in Section 2, MIPs possess specific recognition capacity and superior binding affinity, which could be comparable to natural antibody‐antigen and enzyme‐substrate. In addition, convenient synthesis procedures and diverse polymerization techniques endow MIPs with possibilities to interact with some existing physical sensing method, and enrich the sensing mechanism and signal transduction. These features make MIPs promising materials for novel chemical and biological sensing with desirable functions.^[^ ^220^, ^221^, ^222^ ^]^ In this section, we will give a comprehensive review on the applications of MIPs for chemical and biological sensing, namely MIP‐based sensing, including optical sensing, electrochemical sensing, chemiluminescence (CL) sensing, and some others.

#### 4.2.1 Optical Sensing (Fluorescence)

As is well known, optical sensing techniques as a conventional sensing approach have been developed for decades, and research on MIPs materials’ applications has also become a hot topic. The main working mechanism of MIPs‐based optical sensors is to transfer the optical signals (like the changes of optical properties) into electronic signals, where the optical changes would happen during the recognition and binding events of MIPs materials. Two kinds of MIPs‐based optical sensors have been proposed, including MIP‐affinity optical sensors and MIP‐optoelectronic sensors. MIP‐affinity optical sensors primarily aim at analytes with inherent optical properties, such as refractive index, fluorescence intensity, absorbance, and so on. MIP‐optoelectronic sensors are integrated with MIPs, which consist of functional reagents with optical reporting capability. When the affinity binding process initializes, such reagents could sensitively respond to the surrounding changes with optical variations.

Fluorescence intensity is a kind of typical and commonly used signal of MIP‐based optical sensors, due to the low detection limits, high sensitivity, and convenience. When the target analyte is active fluorescent intrinsically, its chromophore or fluorophore could give rise to the fluorescence intensity changes during MIP binding process for quantitative or qualitative analysis. Recently, numerous MIP‐based fluorescent sensors have been developed. For instance, Moreno‐Bondi et al. designed MIP‐film arrays with nanopatterns by electron beam nanolithography as sensors for template rhodamine 123 (R123).^[^ ^223^ ^]^ They synthesized cross‐linkable linear copolymer poly(methacrylic acid‐co‐2‐methacrylamidoethylmethacrylate) (P(MAA‐co‐MAAEMA)) to be imprinted with R123 as model fluorescent templates. The results demonstrated that such MIP film arrays showed sensitive recognition capacity at nanomolar level concentration with constant fluorescence intensity. Haupt and his coworkers realized direct fluorescent sensing of target fluoroquinolone antibiotic enrofloxacin (ENRO) based on MIP nanoparticles associated with fluorescence polarization measurements.^[^ ^224^ ^]^ The sensing system performed well on selective recognition and high throughput fluorescent screening of three target fluoroquinolones.

**Figure 19:**
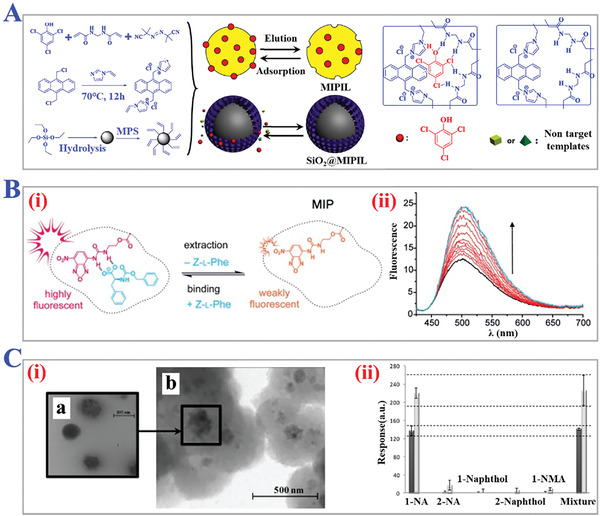
The applications of MIPs‐based fluorescent sensors. A) Scheme of the preparation processes of MIP‐affinity optical sensors for fluorescent detection of 2,4,6‐trichlorophenol. Reproduced with permission.^[^ ^225^ ^]^ Copyright 2020, American Chemical Society. B) (i) Extraction and rebinding of the template switch between high and low fluorescence of prepared highly fluorescent Z‐L‐Phe MIPs. (ii) Fluorescence spectra of MIPs in the absence (black) and presence of Z‐L‐Phe with different concentrations (red and blue). Reproduced with permission.^[^ ^228^ ^]^ Copyright 2013, Wiley‐VCH. C) (i) TEM pictures of super‐paramagnetic hybrid nanoparticles (a) and the magnetic‐MIPs located inside (b). (ii) Optical interference study of 1‐NA imprinted nanoparticles in the presence of individual 1‐NA, 2‐NA, 1‐Naphthol, 2‐Naphthol, 1‐NMA, and mixture. Reproduced with permission.^[^ ^229^ ^]^ Copyright 2011, Elsevier B.V.

However, there are only a few targets with such natural and inherent fluorescence properties (**Figure** **19**A).^[^ ^225^ ^]^ To expand the suitable objects of MIP‐based fluorescent sensors, some approaches have been proposed including labeling templates with fluorescent dyes, replacing original analytes with fluorescent analog derivatives, and choosing fluorescent functional monomers.^[^ ^226^ ^]^ For example, Sellergren et al. designed an automated MIPs‐based competitive assay for on‐line fluorescent sensing of penicillin‐type *β*‐lactam antibiotics (BLAs).^[^ ^227^ ^]^ In the beginning, stoichiometric highly fluorescent competitors (pyrenemethylacetamido penicillanic acid) bearing pyrene labels were packed with analytes in the assay. After the competitive recognition happened, the fluorescence signal was measured relatively with antibiotic concentration of the sample during desorption process. Such a strategy actually enabled the selective sensing of analytes possessing no inherent fluorescence. Besides, Sellergren's group established MIPs microparticles with core‐shell structures as fluorescent sensory of N‐carbobenzyloxy‐L‐phenylalanine (Z‐L‐Phe) (Figure 19B).^[^ ^228^ ^]^ They integrated fluorogenic monomer (nitrobenzoxadiazole (NBD)) into the MIPs matrix shells coated onto the silica core, which acted as urea‐based fluorescent dyes. Upon the analyte binding to the affinity sites of the MIPs shell, the NBD dyes would undertake an intramolecular charge transfer process, leading to an increase of fluorescence, namely “light‐up”. Considering improving the precision of MIP‐based fluorescent sensors, a critical problem demanding prompt solutions is to reduce the interference of background fluorescence noise. Diversifying the topological constructions of resultant MIPs and introducing composite functional materials could be feasible propositions. Valero‐Navarro's group fabricated MIP nanoparticles with topological core‐shell structures for fluorescent sensing of 1‐naphthylamine (1‐NA).^[^ ^229^ ^]^ The MIP nanoparticles were imparted with magnetic core and MIP coating shell by performing precipitation polymerization of *γ*‐Fe_3_O_4_‐oleic acid NPs into poly‐methyl methacrylate‐co‐ethylene glycol dimethacrylate (poly‐MMA‐co‐EDMA). They used a magnetic separator to separate MIP nanoparticles after affinity binding to enrich the fluorescence signal and reduce the background noise of the surrounding matrix, as shown in Figure 19C.

**Figure 20:**
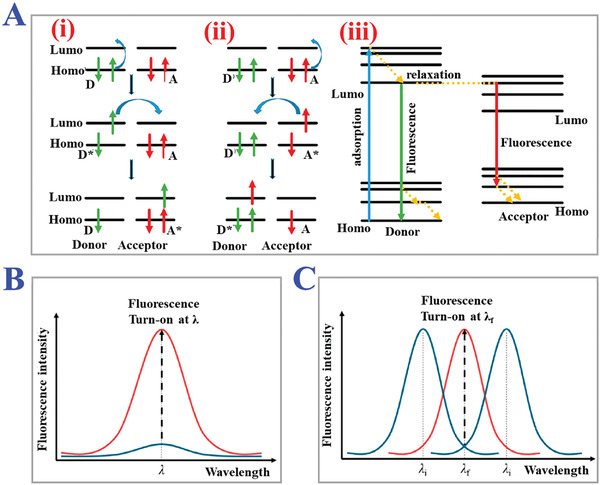
The basic mechanism of QDs‐MIPs‐based fluorescence sensing. A) Fluorescence quenching mechanisms. (i) Photo‐induced reduction mechanism. (ii) Photo‐induced oxidation mechanism. (iii) FRET mechanism. Reproduced under a Creative Commons (CC‐BY) license.^[^ ^232^ ^]^ Copyright 2019, The Authors. Licensee MDPI, Basel, Switzerland. B) Fluorescence intensity enhancement. C) Fluorescence emission wavelength shift. Reproduced with permission.^[^ ^285^ ^]^ Copyright 2013, American Chemical Society.

Apart from the above fluorescence detection methods, MIPs‐based optical sensors on the strength of QDs have also been investigated owing to the intrinsic optical properties like wide absorption spectra, cramped emission spectra, and large stokes shifts (Figure 19C,D).^[^ ^230^, ^231^ ^]^ According to the fluorescence detecting mechanisms, QDs‐MIPs‐based optical sensors could be classified as fluorescence quenching, fluorescence enhancing and fluorescence emission shift (**Figure** **20**A–C).^[^ ^232^ ^]^ The major reason for fluorescence quenching of QDs is photo‐induced electron transfer (PET). The templates would donate excitions for excited QDs by photo‐induced oxidation or accept excitions from excited QDs by photo‐induced reduction. During this transfer of electrons, excited QDs would undergo a relaxation process to the ground state. Dexter electron transfer and fluorescence resonance energy transfer (FRET) would also take place and contribute to the fluorescence quenching. A classical QDs‐MIPs‐based optical sensor is to be designed with core‐shell structure. Tang and his coworkers fabricated Mn‐doped ZnS QDs‐embedded MIPs for phosphorescence sensing of bovine hemoglobin (BHb).^[^ ^233^ ^]^ They used vinyl‐modified Mn‐doped ZnS QDs as supporting core, and then polymerized MIPs layer as the shell through surface graft imprinting. When the analyte BHb gradually bound to the recognition sites in the MIPs shell, the fluorescence intensity of QDs exhibited a trend of respective decrease. Such a congruent relationship would be utilized as the basis of subsequent quantitative sensing. Meanwhile, introducing other quencher like carbon dots (CDs) into MIPs skeleton could be another route. Concretely, Su et al. constructed a ratiometric fluorescent MIPs nanohybrid sensor (CdSe@SiO_2_/CDs) for recognition of 4‐nitrophenol (4‐NP).^[^ ^234^ ^]^ They first prepared CdSe@SiO2 QDs with further modification of organosilane‐functionalized CDs via Si—O bonds, and then sol‐gel molecular imprinting method was employed to form a MIP shell onto the surface. The transfer of fluorescence resonance energy between photoluminescent CDs and template 4‐NP would generate optical response, where the fluorescence intensity of CDs quenched while the QDs’ kept constant. This dual‐emission fluorescent sensing mechanism ensured high sensitivity and detection accuracy.

In contrast to fluorescence quenching, fluorescence enhancing aims to reduce the loss of energy or excitions to an extreme, and interdict the PET or FRET process. As for the fluorescence enhancing mechanism, the Stern–Volmer equation could be applicable to explain its principle:

(1) $$\frac{I_{\max}}{I}=\;1+K_{\mathrm{sv}}\left[C\right]$$

**Figure 21:**
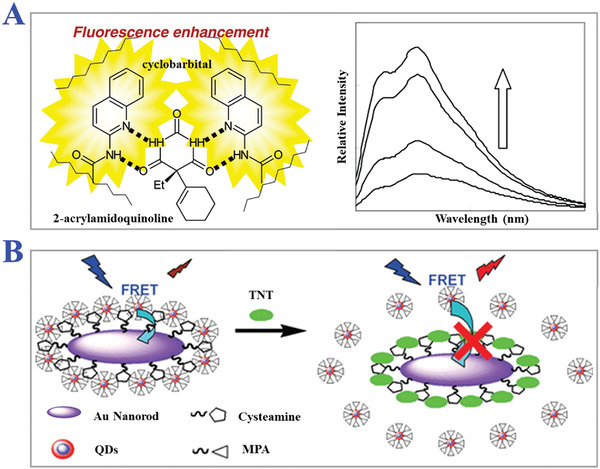
The examples of MIPs‐based fluorescent enhancing principle. A) Introducing 2‐acrylamidoquinoline as a functional monomer for enhancing the fluorescent signal of cyclobarbital imprints. Reproduced with permission.^[^ ^235^ ^]^ Copyright 2005, American Chemical Society. B) Scheme of the FRET blocking‐up process induced by the combination of target TNT. Reproduced with permission.^[^ ^236^ ^]^ Copyright 2011, American Chemical Society.

In this linearized form, *I*_max_ refers to the maximum fluorescence intensity, *I* means the final fluorescence intensity, *K*_sv_ is Stern–Volmer constant which could be calculated by the lifetime of excited state, and [*C*] means the concentration of templates. Above equation could roughly explain the relationship between the fluorescence enhancing ratio and the concentrations of template molecules, though practical circumstance tends to be more complex. We summarized several MIP‐based sensors based on the fluorescence enhancing principle. Kubo’ group realized successful fluorescent enhancing sensing of cyclobarbital via integrating 2‐acrylamidoquinoline as a signaling monomer into MIPs (**Figure** **21**A).^[^ ^235^ ^]^ Upon hydrogen bonding formed between targets and monomers, the PET process of quinoline nitrogen was inhibited and the fluorescence intensity was finally enhanced. Similarly, this strategy is also suitable for the sensory of nonfluorescent compounds based on the detection of raised fluorescence signals. Xia et al. designed a MIP hybrid composed of gold nanorod and CdTe/CdS quantum dots (AuNR‐QDs) as nanosensors for TNT (Figure 21B).^[^ ^236^ ^]^ Through carboxyl‐amine attractive bonding, QDs and AuNR formed a compact assembly, where QDs acted as a donor to transfer energy to acceptor AuNR by FRET principle in the absence of templates. Due to the superior affinity with amine groups, template TNT tended to replace QDs and formed Meisenheimer complexes with amines‐AuNRs. Thus, the FRET architecture was destroyed, and the fluorescence intensity of QDs recovered as the concentration of templates increased.

#### 4.2.2 Electrochemical Sensing

MIP‐based electrochemical sensing, as a universal sensing method for on‐line monitoring target analytes, exhibited distinct functions in the field of drug delivery, pharmaceutical screening, environmental pollutants, food chemistry, and so on.^[^ ^237^, ^238^, ^239^ ^]^ With the development of diverse electrodes, electrochemical sensing showed superior advantages in miniaturization and automation with lower cost of production. A standard strategy for constructing MIP‐based electrochemical sensor is to modify electroactive MIPs nanoparticles or films on the surface of electrode via electro‐polymerization of conductive polymer monomers. The top MIPs layer, electrode, and the bottom transducer were assembled into a “sandwich” structure. There exist three main working patterns of MIP‐based electrochemical sensors. First, when template analytes enter the binding cavity in MIPs, the analytes would undergo a redox reaction with electrode, which generates redox current whose intensity shows a positive correlation with the concentration of analyte. Another mechanism is named as “gate effect”, where redox reactions happen between electrochemical probes and electrodes, and the templates act as gate controllers. In the absence of a template, the electrochemical probes could pass through the MIPs layer, but such a process would be broken if the templates bind to the recognition sites and block the channel, resulting in the decrease of redox current. The last one is competitive mechanism. The analyte derivatives with a similar structure as competitors first bind to the MIPs layer and form an initial REDOX current peak. Such circumstance would be changed after the addition of the template analytes for competitive combination and this change could be recorded as a sensing signal.

##### Construction of MIP‐based Electrochemical Sensor

Generally, two critical steps must be considered in designing a MIP‐based electrochemical sensor: preparation of electroactive MIPs materials, and selection of suitable electrodes with necessary decoration. Given the above studies, suitable electroactive MIPs films or nanoparticles should be synthesized by electro‐polymerization. However, common functional monomers barely bear electrical conductivity. Recently electroactive functional monomers have been an investigation hotspot for enhancing the conductivity of MIPs. For example, Li and his colleagues involved Prussian Blue (PB) into the construction of electrocatalytic MIPs films for the determination of template oxytetracycline (OTC).^[^ ^240^ ^]^ PB, an inorganic conductive substance, served as an electron transfer contributor and electronic media to enhance the electrochemical signals in this competitive sensing system. Meanwhile, some other conductive polymer monomers, such as aniline, thiophene, pyrrole, phenylacetylene, 3,4‐ethylenedioxythiophene (EDOT), etc., have also been employed for improving the electrochemical signals and sensitivity.^[^ ^241^, ^242^ ^]^

Selection and decoration of electrodes are another critical portion because the overall conductivity mainly depends on the intrinsic electrical properties of electrode materials. Noble metals like Au, Pt, Cu, and Ni have been widely chosen to be processed as electrodes due to the large anodic potential range. Some functional groups could be easily modified onto the noble metal film or nanoparticles for subsequent decoration of MIPs layer. Yola et al. designed a novel MIPs‐based electrochemical sensor towards detecting of tyrosine (Tyr).^[^ ^243^ ^]^ The electrode was designed based on cubic gold nanoparticles (cAuNPs) modified with 2‐aminoethanethiol functionalized graphene oxide (2‐AET‐GO), and the results demonstrated that this sensor possessed linear sensitivity, low detection limit, and long‐term reproducibility. However, inevitable metal oxide on the surface of electrodes tends to generate extra background signals and disturb the analysis accuracy. Under this circumstance, carbon‐based electrodes, such as glassy carbon electrodes (GCEs), ceramic carbon electrodes, pencil carbon electrodes, and so on, have become a feasible replacement to overcome this drawback in recent years.^[^ ^244^, ^245^ ^]^ Although the electron transfer rate of carbon is lower than that of noble metal, some other properties like low background current signal and wide potential window could offset the sensing accuracy by sacrificing sectional efficiency. For instance, Ding's group established Au microflowers supported by GCE decorated with dopamine@graphene (DGr) for ultra‐trace determination of cholesterol.^[^ ^246^ ^]^ Polydopamine (PDA) film was used to stabilize Au nanoparticles from aggregation with subsequent immobilization onto the surface of GCE. Such construction actually lowered the background interference both in buffer and human serum samples.

##### Electrochemical Sensing Signals

According to the forms of response, signals of MIPs‐based electrochemical sensors could be classified into four categories: current, potential, conductivity, and capacitance. Such signals are generated during the reactive combination between targets templates and the reaction sites on the electrodes. Among these four types, electric current signals are often characterized by voltammetry, including CV, linear sweep voltammetry (LSV), differential pulse voltammetry (DPV), square wave voltammetry (SWV), etc.^[^ ^247^, ^248^, ^249^ ^]^ Luo et al. proposed a facile strategy where MIPs were grafted on the surface of graphene sheet for electrochemical sensing of 4‐nitrophenol (4‐NP).^[^ ^250^ ^]^ The graphene sheet was immobilized with 4‐vinylcarbazole for guiding the selective polymerization of MIPs reagents. They combined CV and DPV methods to characterize the electrochemical behavior during the recognition of target templates 4‐NP. The measured DPV current of such electrochemical sensor was proved to be much shorter, and the current peak responded sensitively and accurately to the 4‐NP concentration with linearly proportional relationship. To generate a current signal strong enough, the components of electrochemical sensors must be electroactive, otherwise the responsive signals will not be detectable, which restricts the wider applications of electric current‐based MIPs sensors for non‐electroactive analytes or ramifications.

**Figure 22:**
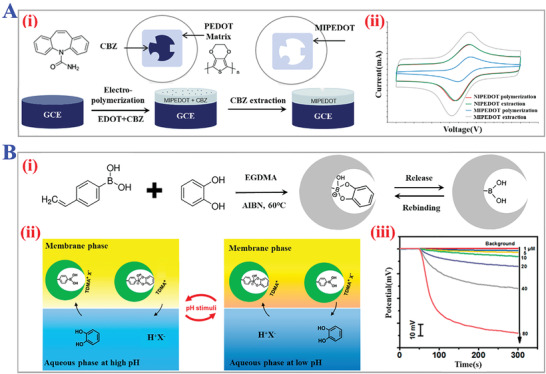
Electric current and potentiometry signals‐based MIPs‐electrochemical sensors. A) (i) Schematic fabrication of carbamazepine‐imprinted PEDOT GCE. (ii) CV signals of several experimental groups transduced by MIPs electrode. Reproduced with permission.^[^ ^252^ ^]^ Copyright 2021, Elsevier B.V. B) (i) Synthesis process of pH‐responsive boronate‐affinity MIPs for potentiometric sensor. (ii) Response and regeneration mechanisms of such electrochemical sensor. (iii) Dynamic potential response profiles of the pH‐responsive MIP‐based electrode. Reproduced with permission.^[^ ^253^ ^]^ Copyright 2020, American Chemical Society.

Potentiometry is another method to record the potentials of targets‐rebound electrodes during electronic reactions. Ion‐selective electrodes (ISEs) are considered as top‐priority for potentiometry, which uses a special electrode membrane that selectively responds to specific ions which serve as templates. When ISEs contact with the solution containing target ions, membrane potential would be generated at the phase interface between ISEs’ sensitive membrane and the solution related directly to the ion activity. Owing to good selectivity and short equilibrium time, ISEs have become the most commonly used indicator electrode for potential analysis based on MIPs. Another choice for potentiometry is extended‐gate field‐effect transistors (EG‐FETs), a voltage‐controlled semiconductor device. Without complex electronic instruments, EG‐FETs are capable of realizing dynamic changes of potential at regular intervals, which could be converted to detectable potential signals. Based on the advantages of the two potentiometry methods, Noworyta's group constructed a novel electrochemical sensor for sensing renal disfunction biomarkers by combining MIPs film and EG‐FETs.^[^ ^251^ ^]^ They coated the inosine‐imprinted MIPs film on the surface of EG‐FET via electrochemical polymerization. As expected, the EG‐FET, as a signal transducing unit, acted well during the template recognition process with a linear dynamic concentration range and high detectability. What's more, the adjustment capacity of gate voltage actually imparted this electrochemical sensor with flexibility for multiple practical demands. Some other concrete applications of electro‐MIPs sensors on the basis of current and potential signals have been developed. For instance, Hammoud et al. fabricated a new MIPs‐based electrochemical sensor consisting of carbamazepine (CBZ)‐imprinted PEDOT layer on the surface of GCE, as presented in **Figure** **22**A.^[^ ^252^ ^]^ They adopted CV method to synthesize molecular imprints for high sensitivity and selectivity. [Fe(CN)_6_]^3‐/4‐^ was chosen as redox pair/mediator for subsequent CV and SWV tests of electrodes’ signals. The results actually proved that such MIPs sensor freed up more polymer sites and increased the signal‐to‐noise ratio. Liang and his colleagues proposed MIPs‐based potentiometric sensor with stimulus‐responsiveness for reversible detection of neutral phenols (Figure 22B).^[^ ^253^ ^]^ Such sensor was constructed based on the MIPs polymeric membrane copolymerized by model catechol and 4‐vinylphenylboronic acid (4‐VPBA) as pH‐responsive monomer. It was demonstrated that binding sites would be regenerated via external stimulus of weakly alkaline aqueous solution after each measurement, which could be repeated up to five‐six sensing cyclic. Dynamic potential responses were also measured stably to catechol and some analogs numerous times. This smart electrochemical MIPs‐based sensor was imparted with the flexibility of incorporating different responsive properties like pH, photonic and thermal, and such an MIP‐based sensing strategy broadened reversible electrochemical sensory of various organic and biological species.

**Figure 23:**
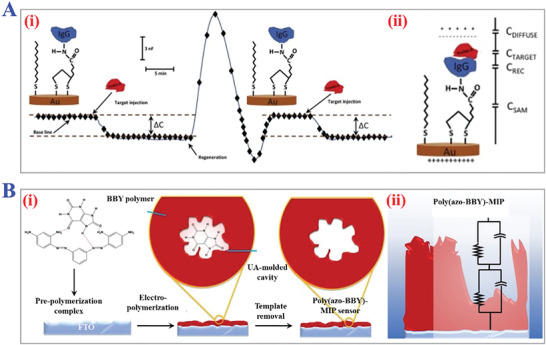
Conductivity and capacitance signals‐based MIPs‐electrochemical sensors. A) (i) Schematic diagram of the variation in capacitance (ΔC) as a function of time during binding between anti‐human IgG and protein A. (ii) Immobilization of the receptor molecule on the transducer surface via self‐assembled monolayer of alkylthiols. Reproduced with permission.^[^ ^286^ ^]^ Copyright 2015, Elsevier B.V. B) (i) Synthesis procedure of the poly(azo‐BBY)‐MIP sensor platform for detection of uric acid. (ii) Schematic representation of impedance circuit models of poly(azo‐BBY)‐MIP. Reproduced with permission.^[^ ^255^ ^]^ Copyright 2021, Elsevier B.V.

Sensing of conductivity and capacitance signals are primarily based on the MIPs films or membranes. According to the electrical double‐layer theory, there exists potential at the electrode‐electrolyte interface and double electrical double layer. The electrode phase would concentrate excess charges on its surface, while electrolyte with large resistance would only concentrate closely to the phase interface, namely tight double layer. The electronic activation reactions and adsorption process only take place in the tight double layer. Hence the MIPs membranes which performed the role of insulative electrolyte should be fabricated with excellent electrical insulativity, where the templates undergo a recognition process, as shown in **Figure** **23**A.^[^ ^286^ ^]^ We enumerated several MIPs sensors monitoring conductivity and capacitance signals. For example, Ni and his coworkers established BPA sensor comprising of Titanium nitride‐reduced graphene oxide (TiN‐rGO) composite and core‐shell MIPs.^[^ ^254^ ^]^ They coated pyrrole with electrical conductivity on silica and carbon paste electrode‐contained TiN‐rGO was confirmed to be improved. The novel sensor performed a wide range of linear responses and low detection limit, and showed great potential in environmental analysis. In addition, Teixeria's group designed uric acid‐imprinted electrochemical platform, as presented in Figure 23B.^[^ ^255^ ^]^ The azo group of the polymers provided quantifiable redox capacitance of the electrode surface equipped with EIS or electrochemical capacitive spectroscopy (ECS). Most important was that this sensor did not depend on an extra soluble redox probe and the limit of detection was successfully lowered to 0.160 µmol L^−1^. Dickert et al. proposed a conductometric MIPs sensor for monitoring engine oil.^[^ ^256^ ^]^ They used sol‐gel polymerization method to prepare aminopropyl‐triethoxysilane (APTES) imprinted layers as recognition units, and thin‐film gold electrodes acted as transducer units. Furthermore, polyurethane layers were modified with multi‐walled carbon nanotubes as conductive fillers for the improvement of electrical conductance signals. The results depicted that this sensor showed superior sensitivity over a wide concentration range of analyte oil. Yao's team designed MIPs‐based capacitive sensor for the specific determination of tegafur.^[^ ^257^ ^]^ Gold capacitance electrode was first coated with m‐aminophenol, and then they realized the insulation of sensitive membrane by controlling a smaller potential scan rate during electrochemical polymerization rather than treating it with alkanethiol. Electrochemical impedance (EI) experiment successfully identified the enhanced sensitivity with a shorter response time. Generally, MIPs‐based conductometric and capacitive sensors have gradually evoked interest due to their advantages of easy operations, on‐line dynamic monitoring, highly sensitive, and so on.^[^ ^258^, ^259^ ^]^ However, some drawbacks like shielding interference signals, synthesis of thinner MIPs membranes and closer packing still restricted their further development.

#### 4.2.3 Electrochemiluminescence (ECL) Sensing

Electrochemiluminescence (ECL) sensing is a promising technique combining electrochemical reactions and CL method. In a typical ECL procedure, various electrodes were employed to perform electrical input like voltage or current to an electrical system containing chemiluminescent substances, and activated electronic reactions would supply sufficient energy for such chemiluminescence transition towards an excited state. With gradual relaxation to ground state, emitted light could be recorded by an optical fiber or amplified via photomultiplier for subsequent trace analysis of analytes. However, nowadays available ECL sensors mostly suffered from the dilemma of weak anti‐interference capacity due to the terrible specificity. Thus, the introduction of MIPs into ECL sensors is a reliable approach to enhance the selectivity and recognition specificity of the electronic reaction, and reduce the interference signals. For instance, Luo and his colleagues designed an ECL sensor for the sensitive determination of Ribonuclease A (RNase A).^[^ ^260^ ^]^ The sensor was constructed by coating RNase A‐imprinted MIPs layer on the surface of the Fe_3_O_4_/multi‐wall carbon nanotubes/SiO_2_ support. To examine the anti‐interference ability of the sensor, several foreign substances were employed, and the results confirmed the positive impact of MIPs grafting on improving detection selectivity of ECL. Gan et al. established a novel ECL immunosensor with a single antibody sandwich for trace sensing of protein hemoglobin (Hb) based on magnetic MIPs.^[^ ^261^ ^]^ They imprinted MIPs with template Hb and labeled antibodies with Ru‐silica (Ru(bpy)_3_^2+^‐doped silica) doped Au (Ru@SiO_2_@Au) nanocomposites as signal tags. Such composite could amplify the ECL intensity with the enrichment function of magnetic MIPs, which contributed to ultrasensitivity and lower detection limit, where the logarithm of ECL intensity was linearly relative to Hb concentration.

#### 4.2.4 Other Sensing

Except for the above typical sensing methods, researchers have also developed some other MIPs‐based sensing techniques with diverse novel mechanisms, such as combination with surface‐enhanced Raman scattering (SERS) technology and Surface plasmon resonance (SPR) technology.^[^ ^262^, ^263^ ^]^ These neoteric sensing techniques enriched the existing MIPs‐based sensing systems and advanced MIPs sensors for wider chemical and biological applications.

##### Surface‐Enhanced Raman Scattering (SERS) Sensing

Benefitting from the advantages of fingerprint recognition, rapid responsiveness, and nondestructive test, SERS sensing has been considered as an ultra‐sensitive vibrational spectroscopy technique for biological sample detection. Similar to other MIPs‐based sensors, MIP‐SERS sensors are mainly composed of MIPs layer and SERS substrate. The MIPs layer renders the sensor with a strong affinity to target analytes, and upon the targets entering the imprinted cavities, SERS signals would be generated under the activation of incident light. There are two primary sensing modes of MIP‐SERS sensors: label‐free mode and SERS probe mode. The label‐free mode is suitable for analytes with inherent Raman signals. SERS probe is another protocol proposed for sensing those substances with weak Raman signals. For example, Holthoff's team reported a new MIP‐SERS sensor for detecting TNT.^[^ ^264^ ^]^ They deposited thin MIPs films that were constituted by sol‐gel‐derived xerogels as TNT imprints on a SERS‐active surface. During the binding process of TNT in MIPs matrix, a SERS band appeared with a characteristic Raman shift peak at the position of ≈830 and ≈1350 cm^−1^. This sensor exhibited a reversible response to target TNT with a 3 µm detection limit and excellent stability in different environments. Meanwhile, combined with different MITs, MIP‐SERS sensors could be modified to have higher sensitivity, intelligent detection, and wider analytes (micromolecules, macromolecules, proteins, etc.). In the last decades, multifunctional monomers and environmental‐responsive polymers have been a hotspot for the investigation of MIPs, and abundant thermo‐responsive, magnetic‐responsive, or ion‐responsive MIPs were applied for constructing MIP‐SERS sensors. Yan et al. established a thermoresponsive MIP‐SERS sensor for rhodamine 6G (R6G) detection in water samples.^[^ ^265^ ^]^ The sensor was designed with a layer of NIPAM‐based MIPs coated on the ZnO/Ag SERS substrate via precipitation polymerization. When the temperature of surrounding environment reached an optimal 30 °C, the imprints cavities within MIPs would transform to the best shape for completely binding with R6G. Under this circumstance, the peaks of Raman signals, which appeared extremely weak at other temperatures, could be detected. Generally, the combination with novel MIPs really expands SERS sensors’ practical applications in the fields of chemo/bioanalysis.

##### Surface Plasmon Resonance (SPR) Sensing

SPR sensing technique has been extensively employed for biological sensing of biomolecular recognition with superiority of label‐free detection, no extra damages, needless pre‐purification, and real‐time tracing. On the surface of metal medium, there exists an electronic gas medium layer comprising of positive and negative charges with high density, namely surface plasmon (SP). When biological reactions occur at the metal‐dielectric interface, the refractive index of the medium would vary and SP waves would dissipate the energy of incident photon, thus leading to the relevant decay of reflected light intensity at a specific incident angle.^[^ ^266^, ^267^ ^]^ By decorating a thick MIPs layer on the metal surface, a metal‐dielectric interface could be formed, benefiting from which the recognition efficiency of biomolecules and resonance signals could be improved and enhanced. A promising solution for constructing MIPs‐based SPR sensor is in situ polymerization of MIPs on metal SPR platforms via bottom‐up strategy, and MITs such as photo‐induced polymerization, RAFT polymerization, ATPR polymerization, and live radical polymerization have been involved. Syritski's team synthesized a protein‐selective MIPs film by controlled/living radical polymerization on the surface of gold SPR sensor.^[^ ^268^ ^]^ Such in situ induced polymerization method realized compact covalent attachment with the gold substrate, and the biomolecules rebinding process could be sensitively detected. Some interferent proteins were also adopted to evaluate the recognition specificity and the MIP‐SPR sensor showed no responsive signals.

Apart from the above bottom‐up strategy, top‐down fabrication was another route to deposit MIPs onto the SPR surface from prep‐polymerized solutions. For instance, Lautner et al. proposed a new concept to prepare MIP‐SPR sensors for protein determination via standard photolithographic technology.^[^ ^269^ ^]^ They first produced avidin‐imprinted conducting polymer microbands comprising of poly(3,4‐ethylenedioxythiophene)/poly(styrenesulfonate) (PEDOT/PSS), and then successfully deposited them on bare Au SPR chips directly. This top‐down concept made MIP‐SPR sensors easier to manufacture and straightforward to sense. What's more, the great achievements of responsive MIPs have also promoted the development of the MIP‐SPR sensors with lower detection limits and more sensitive SPR signals. Sugimoto and his coworkers designed Au NPs‐embedded MIP‐SPR sensor chip for sensing target dopamine with low molecular weight species.^[^ ^270^ ^]^ The Au NPs could selectively combine with target small molecules during the swelling process of the MIPs layer. More importantly, the distances between Au NPs embedded in the MIPs were enlarged, resulting in the increase of SPR angle shift with enhanced SPR reflectance intensity.

### 4.3 MIPs for Gravimetric Analysis

**Figure 24:**
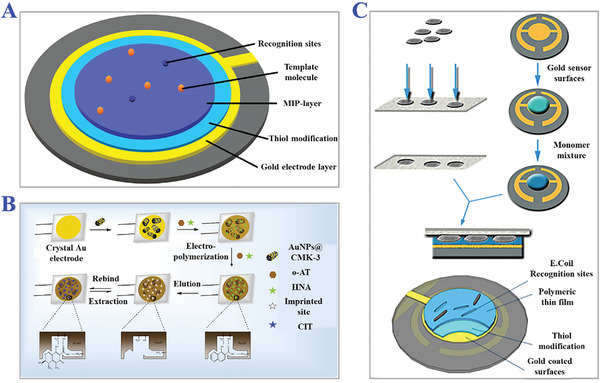
The applications of MIPs for gravimetric analysis. A) Typical structure of MIP‐based QCM analyzer. Reproduced under a Creative Commons (CC‐BY) license.^[^ ^232^ ^]^ Copyright 2019, The Authors, Creative Commons (CC‐BY) license. Licensee MDPI, Basel, Switzerland. B) Fabrication scheme of MIP‐QCM substrate based on Au@mesoporous carbon CMK‐3 (Au@CMK‐3) as functional composite for ultrasensitive determination of CIT. Reproduced with permission.^[^ ^273^ ^]^ Copyright 2016, Elsevier B.V. All rights reserved. C) Illustration of the construction of *E. coli*‐imprinted QCM platform. Reproduced with permission.^[^ ^274^ ^]^ Copyright 2014, Elsevier B.V.

The change of mass during chemical or biological reactions could serve as a quantitative sensing index, namely gravimetric sensing. Nowadays gravimetric sensing methods are mainly based on the principle of piezoelectric effect and the frequency variation of QCM element.^[^ ^232^, ^271^, ^272^ ^]^ Typical QCM sensors could sensitively detect the extremely tiny mass changes occurring on the surface of QCM electrode, but their sensing specificity could be hardly controlled. The recognition selectivity of integral sensor could be improved by decorating a layer of MIPs as functional units on the electrode. For better coating of functional MIPs layers, the surface of QCM electrodes could be modified by Thiol, imparting them with sufficient sulfydryl groups (**Figure** **24**A).^[^ ^232^ ^]^ According to the Sauerbrey equation:

(2) $$\Delta f\;=\;-2.6\times 10^{6}f_{0}^{2}\Delta m/A$$

where Δ*f* and *f*_0_ refer to the changeable and resonant frequency of the QCM electrode, respectively, Δ*m* is the mass change during the biological reactions, and *A* means the piezoelectrically active crystal area. When the analytes entered the cavity of MIPs layer, the mass change could be calculated by detecting the value of Δ*f*. Plenty of studies have designed diverse MIP‐QCM sensors for the determination of different types of analytes like bio‐micromolecules or macromolecules including proteins, viruses, and even cells.^[^ ^273^, ^274^ ^]^ Lin and his coworkers developed QCM sensor decorated with thin proteins‐imprinted MIPs films for sensing three digestive proteins in saliva.^[^ ^275^ ^]^ Poly(ethylene‐co‐vinyl alcohol) (PEVAL) was used to form the coating layer via the thermally induced phase separation method, and the noncovalent recognition effectiveness could yield to highest by tuning the ethylene mole ratios of PEVALs. The detection accuracy of the targets’ concentrations converted from QCM frequency signals exceeded 90%, which was superior to commercial instruments. Furthermore, MIP‐QCM sensors could be integrated with electrochemical methods. Such sensors have similar structures to MIP‐based electrochemical sensors, where the substrates are replaced with QCM electrodes. Fang and his colleagues described a novel QCM sensor modified with 3D MIPs composite for trace detection of citrinin (CIT), as shown in Figure 24B.^[^ ^273^ ^]^ They applied electro‐polymerization method to form MIPs membrane containing o‐aminothiophenol onto the surface of Au electrode decorated with Au nanoparticles@mesoporous carbon CMK‐3 (AuNPs@CMK‐3). Under optimal conditions, a linear relationship between the frequency shift of QCM and the CIT concentrations as the sensory formula was achieved with a relatively low detection limit. Such MIP‐QCM sensors integrated with electrochemical handling procedure showed superior anti‐interference ability, chemical stability, and satisfactory recoveries. Debizli et al. applied label‐free QCM sensors for the detection of pathogenic bacteria (*E. coli*) in water samples (Figure 24C).^[^ ^274^ ^]^ The whole *E. coli* bacteria was imprinted on QCM mass‐sensitive devices via micro contact imprinting method, and N‐methacryloyl‐L‐histidine methylester was employed to construct similar recognition ability to natural antibodies. This study offered a rapid and sensitive method to detect bacteria, and paved the way for integrating the imprinting of whole cell or bacteria into MIPs sensors.

### 4.4 MIPs for Point‐of‐Care Testing (POCT) Platform

As a typical mechanism employed for point‐of‐care testing (POCT), colorimetric method is commonly combined with photonic crystal (PhCs) materials, which possess brilliant structural colors. Due to the periodically ordered spatial structure, PhCs form photonic band gap (PBG) where only light of particular wavelength could diffuse. Therefore, PhCs have unique reflection wavelengths that can be used as a colorimetric sensing index.^[^ ^276^, ^277^, ^278^ ^]^ Nevertheless, natural PhCs are suitable for direct integration with MIPs because of their robust hardness, insufficient components, and chemical inertness. Inspired by such amazing structures of natural PhCs, artificial inverse opal structure could be easily replicated via hydrogel polymers, and applied for the construction of MIPs colorimetric sensors. Recently, Zhao's group developed a novel multiple‐targets platform by imprinting three template biomolecules into the inverse opal hydrogel skeleton (creatine and urea) and on the surface of the consecutive cavities (lysozyme) separately.^[^ ^279^ ^]^ During the binding process of target molecules, the average refractive index of the integral hydrogel skeleton gradually changed and the structural colors changed correspondingly. By monitoring the shift values of characteristic reflection peak, the recognition progress could be detected as colorimetrical signals. Moreover, this sensing strategy could be nearly influenced by external surroundings or interferents thanks to such inherent and stable properties of PhCs, assuring precision and sensitivity. In brief, the critical part of fabricating colorimetric MIPs platform is to impart the MIPs substrate with characteristic color properties, whether they are from intrinsically structural or just containing colored complexes. Barsbay et al. prepared a MIPs colorimetric platform by performing thermally‐initiated RAFT‐mediated copolymerization.^[^ ^280^ ^]^ They coated benzophenone onto the surface of poly(ethylene terephthalate) (PET) to induce the RAFT grafting polymerization activated by the homolytic cleavage, and finally achieved a MIP layer with a thickness of just 100 nm. It was proved that the target textile dye BR9 was specifically captured by the MIP film, with an obvious color change from white to magenta. Additionally, a smartphone was utilized for on‐line colorimetric analysis of practical samples. This strategy endows colorimetric MIPs platforms with merits of miniaturization and portability, and broadens the development prospects in the field of POCT.^[^ ^281^, ^282^, ^283^, ^284^ ^]^

## 5 Conclusion and Perspective

In this review, we have summarized recent research progress on the bio‐inspired imprinting materials, namely, MIPs, ranging from the fundamental mechanisms, and numerous MITs to the practical applications of sample separation and sensing. Bearing the superiorities like specific affinity, highly sensitive, and recognition efficiency against nature receptors, it was demonstrated that MIPs had promising application potentials in the field of biomedical science, like sample pretreatment, analyte extraction, sensing, drug delivery, catalysis, food chemistry, environment monitoring, etc.

Nowadays, the theories for specific interactions in nature have gone deep into the level of molecules. With further physical investigations on atomic level, interactions between atoms also contribute to more precise explanations for such specific recognition, construction of molecular imprints, and retainable rebinding affinity. More natural interactions existing in natural organisms like specific recognition of transporters in human signaling pathways or the recognition affinity of some biological individuals to certain substances could be learned to enrich the theoretical explanation of mechanisms of molecular imprinting. The study of thermodynamics and spatial configurations of template molecules during removal and rebinding processes also contribute to revealing the deeper principles of molecular imprinting technologies.

In spite of so amazing performance and advancements, there still exist some bottleneck drawbacks to be addressed, such as insufficient theoretical research on imprinting mechanisms, imprinting of biological macromolecules (e.g., whole living cells or even larger biological complex), selection of suitable templates and multiple binding sites. The complicated and bulky configuration of biological macromolecules would lead to weak mobility within cross‐linked networks, and the critical functional sites would be easily sheltered. Besides, water‐solubility would hinder the immobilization of monomers during conventional polymerization process. Strategies like integrating surface imprinting or microcontact imprinting method with enhanced hydrophobic interaction could be feasible to realize imprinting of biological macromolecules.

Meanwhile, choosing suitable templates should not be ignored during the fabrication of MIPs. Briefly, detectable analytes in samples are mainly selected as templates. However, considering the synthesis cost, safety, and chemical instability, not all components could be pre‐combined as imprints in the polymer matrix, which inevitably results in leakage of suitable templates. To solve this problem, employing dummy templates or functional fragments as a replacement can remit this dilemma. Given this, more efforts should be paid to sort suitable substitutes under the premise of ensuring enough recognition specificity and binding efficiency to initial templates. Another issue is in regard to the construction of multiple binding sites. To form one kind of specific binding site, only one template could link with its corresponding functional monomer. Multiple binding sites mean heterogeneous template‐monomer complexes involved with more possibility of mixing non‐selective sites, which further weakens the specific recognition capacity of resultant MIPs. As alternative solutions, semi‐covalent imprinting methods, sacrificial spacer method, sol‐gel process, and so on have served to control the bond length and well‐define the distribution of multiple binding sites.

In addition, the practical application of imprinted materials could be widely explored. Nowadays numerous imprinted materials have been reported with diverse excellent properties in the laboratory. However, when applied for particle and complex samples, such materials could not perform as well as anticipated due to the inevitable interference of impurities, uncontrollable environmental conditions, and so on. Therefore, integrating imprinted materials with some other functional modules into a whole system is a promising route. Considering the requirements of specific applications, the imprinted materials could be packed as a universal dismountable or portable unit which could cooperate with other units undertaking different functions just through unified interfaces. Such imprinted materials‐involved systems could be introduced into more practical scenarios like auxiliary clinical diagnosis and therapy, construction of medical instruments, constitution of living biomaterials, and so on, which surpass beyond typical applications in detection science and analysis technology.

In conclusion, bio‐inspired imprinting materials actually have attained remarkable achievements as extraction media, artificial antibodies, and intelligent sensors for purification and determination of chemical‐biological samples in practical applications. Future investigations and attempts should concentrate on the exploration of molecular imprinting mechanisms at the microscopic scale, and more advanced instruments should be integrated for a more elaborate characterization of the recognition process to remedy the drawbacks of MIPs. Besides, seeking novel polymerization methods with wider applicable imprinting targets and broadening wider fields of applications also deserve more attention. We expect this comprehensive review will give birth to new sparkles for more breakthroughs in bio‐inspired imprinting materials.

## Conflict of Interest

The authors declare no conflict of interest.

## References

[1] M. V. Polyakov , Zh. Fiz. Khim. 1931, 2, 799.

[2] O. Ramström , K. Mosbach , Curr. Opin. Chem. Biol. 1999, 3, 759. 10600723 10.1016/s1367-5931(99)00037-x

[3] S. J. Li , S. S. Cao , M. J. Whitcombe , S. A. Piletsky , Prog. Polym. Sci. 2014, 39, 145.

[4] J. X. Wang , H. Qiu , H. Q. Shen , J. M. Pan , X. H. Dai , Y. S. Yan , G. Q. Pan , B. Sellergren , Biosens. Bioelectron. 2016, 85, 387. 27208472 10.1016/j.bios.2016.05.041

[5] S. C. Zimmerman , N. G. Lemcoff , Chem. Commun. 2004, 1, 5. 10.1039/b304720b 14737309

[6] X. B. Luo , H. Y. Yu , Y. Xi , L. L. Fang , L. L. Liu , J. M. Luo , RSC Adv. 2017, 7, 25811.

[7] G. D. Olssona , B. C. G. Karlssona , S. Shoravia , J. G. Wiklandera , I. A. Nicholls , J. Mol. Recognit. 2012, 25, 69. 22290767 10.1002/jmr.2147

[8] Y. Q. Lv , T. W. Tan , F. Svec , Biotechnol. Adv. 2013, 31, 1172. 23466364 10.1016/j.biotechadv.2013.02.005

[9] M. Haruki , Y. Konnai , A. Shimada , H. Takeuchi , Biotechnol. Prog. 2007, 23, 1254. 17672479 10.1021/bp070130c

[10] Z. Y. Chen , Z. D. Hua , L. Xu , Y. Huang , M. P. Zhao , Y. Z. Li , J. Mol. Recognit. 2008, 21, 71. 18247357 10.1002/jmr.870

[11] L. X. Chen , X. Y. Wang , W. H. Lu , X. Q. Wu , J. H. Li , Chem. Soc. Rev. 2016, 45, 2137. 26936282 10.1039/c6cs00061d

[12] G. Vasapollo , R. D. Sole , L. Mergola , M. R. Lazzoi , A. Scardino , S. Scorrano , G. Mele , Int. J. Mol. Sci. 2011, 12, 5908. 22016636 10.3390/ijms12095908 PMC3189760

[13] L.i. X. Chen , S. F. Xu , J. H. Li , Chem. Soc. Rev. 2011, 40, 2922.21359355

[14] V. B. Kandimalla , H. Ju , Anal. Bioanal. Chem. 2004, 380, 587. 15480581 10.1007/s00216-004-2793-9

[15] H. R. Culver , N. A. Peppas , Chem. Mater. 2017, 29, 5753. 30880872 10.1021/acs.chemmater.7b01936 PMC6420229

[16] K. Mosbach , K. Haupt , J. Mol. Recogn. 1998, 11, 62. 10.1002/(SICI)1099-1352(199812)11:1/6<62::AID-JMR391>3.0.CO;2-5 10076808

[17] G. Wulff , Angew. Chem., Int. Ed. Engl 1995, 34, 1812.

[18] M. Yoshikawa , K. Tharpa , Ş. O. Dima , Chem. Rev. 2016, 116, 11500. 27610706 10.1021/acs.chemrev.6b00098

[19] A. G. Mayes , M. J. Whitcombe , Adv. Drug Delivery Rev. 2005, 57, 1742. 10.1016/j.addr.2005.07.011 16225958

[20] A. Poma , A. P. F. Turner , S. A. Piletsky , Trends Biotechnol. 2010, 28, 629. 20880600 10.1016/j.tibtech.2010.08.006

[21] F. Lanza , B. Sellergren , Chromatographia 2001, 53, 599.

[22] S. Al‐Kindy , R. Badía , J. L. Suárez‐Rodríguez , M. E. Díaz‐García , Crit. Rev. Anal. Chem. 2007, 30, 291.

[23] C. Alvarez‐Lorenzo , A. Concheiro , J. Chromatogr. B 2004, 804, 231. 10.1016/j.jchromb.2003.12.032 15093177

[24] J. O. Mahonya , K. Nolana , M. R. Smytha , B. Mizaikoff , Anal. Chim. Acta 2005, 534, 31.

[25] M. Schiek , F. Balzer , K. Al‐Shamery , J. R. Brewer , A. Lützen , H. G. Rubahn , Small 2008, 4, 176. 18203230 10.1002/smll.200700483

[26] M. J. Whitcombe , E. N. Vulfson , Adv. Mater. 2001, 13, 467.

[27] C. Alexander , H. S. Andersson , L. I. Andersson , R. J. Ansell , N. Kirsch , I. A. Nicholls , J. O'Mahony , M. J. Whitcomb , J. Mol. Recognit. 2006, 19, 106. 16395662 10.1002/jmr.760

[28] O. Ramstrom , R. J. Ansell , Chirality 1998, 10, 195.

[29] D. L. Huang , R. Z. Wang , Y. G. Liu , G. M. Zeng , C. Lai , P. Xu , B. A. Lu , J. J. Xu , C. Wang , C. Huang , Environ. Sci. Pollut. Res. 2015, 22, 963. 10.1007/s11356-014-3599-8 25280502

[30] L. Ye , Adv. Biochem. Eng./Biotechnol. 2015, 150, 1. 10.1007/10_2015_313 25840705

[31] G. Ertürka , B. Mattiasson , J. Chromatogr. B 2016, 1021, 30. 10.1016/j.jchromb.2015.12.025 26739371

[32] Y. M. Zhang , J. Zhang , Q. J. Liu , Sensors 2017, 17, 1567.

[33] R. N. Chen , S. H. Kang , J. Li , L. N. Lu , X. P. Luo , L. Wu , Anal. Methods 2021, 13, 4538. 34570126 10.1039/d1ay01014j

[34] K. G. Yang , S. W. Li , L. K. Liu , Y. W. Chen , W. Zhou , J. Q. Pei , Z. Liang , L. H. Zhang , Y. K. Zhang , Adv. Mater. 2019, 31, 1902048. 10.1002/adma.201902048 31423663

[35] M. Komiyama , T. Mori , K. Ariga , Bull. Chem. Soc. Jpn. 2018, 91, 1075.

[36] L. Ye , K. Haupt , Anal. Bioanal. Chem. 2004, 378, 1887. 15064898 10.1007/s00216-003-2450-8

[37] J. E. Lofgreen , G. A. Ozin , Chem. Soc. Rev. 2014, 43, 911. 24247659 10.1039/c3cs60276a

[38] L. Bettada , H. Y. Tsai , C. B. Fuh , Nanomaterials 2022, 12, 411. 35159755 10.3390/nano12030411 PMC8840370

[39] J. Kalecki , Z. Iskierko , M. Cieplak , P. S. Sharma , ACS Sens. 2020, 5, 3710. 33225686 10.1021/acssensors.0c01634 PMC7771019

[40] S. C. Liu , J. M. Pan , H. J. Zhu , G. Q. Pan , F. X. Qiu , M. J. Meng , J. T. Yao , D. Yuan , Chem. Eng. J. 2016, 290, 220.

[41] S. Al‐Kindy , R. Badia , M. E. Diaz‐Garcia , Anal. Lett. 2002, 35, 1763.

[42] Y. P. Huang , Z. S. Liu , C. Zheng , R. Y. Gao , Electrophoresis 2009, 30, 155. 19072928 10.1002/elps.200800410

[43] Y. Zhang , D. W. Zhang , H. L. Liu , Polymers 2019, 11, 708.

[44] K. Puzio , R. Delepee , R. Vidal , L. A. Agrofoglio , Anal. Chim. Acta 2013, 790, 47. 23870408 10.1016/j.aca.2013.06.036

[45] N. Murase , S. I. Taniguchi , E. Takano , Y. Kitayama , T. Takeuchi , Macromol. Chem. Phys. 2015, 216, 1396.

[46] M. Nomachi , T. Kubo , K. Hosoya , K. Kaya , Anal. Bioanal. Chem. 2006, 384, 1291. 16501954 10.1007/s00216-006-0310-z

[47] T. H. Nguyena , R. J. Ansell , J. Mol. Recognit. 2011, 25, 1.

[48] M. E. Davis , A. Katz , W. R. Ahma , Chem. Mater. 1996, 8, 1820.

[49] G. Wulff , K. Knorr , Bioseparation 2001, 10, 257. 12549870 10.1023/a:1021585518592

[50] J. P. Rosengren‐Holmberg , J. G. Karlsson , J. Svenson , H. S. Andersson , I. A. Nicholls , Org. Biomol. Chem. 2009, 7, 3148.

[51] P. P. Qi , J. C. Wang , L. D. Wang , Y. Li , J. Jin , F. Su , Y. Z. Tian , J. P. Chen , Polymer 2010, 51, 5417.

[52] P. Curcio , C. Zandanel , A. Wagner , C. Mioskowski , R. Baati , Macromol. Biosci. 2009, 9, 596. 19434676 10.1002/mabi.200900056

[53] K. P. Prathish , V. Vishnuvardhan , T. P. Rao , Electroanalysis 2009, 21, 1048.

[54] N. W. Turner , C. W. Jeans , K. R. Brain , C. J. Allender , V. Hlady , D. W. Britt , Biotechnol. Prog. 2006, 22, 1474. 17137293 10.1021/bp060122g PMC2666979

[55] X. T. Shen , L. H. Zhu , N. Wang , L. Ye , H. Q. Tang , Chem. Commun. 2012, 48, 788. 10.1039/c2cc14654a 22139426

[56] C. Dupont , D. R. Armant , C. A. Brenner , Semin. Reprod. Med. 2009, 27, 351. 19711245 10.1055/s-0029-1237423 PMC2791696

[57] S. N. He , L. P. Zhang , S. K. Bai , H. Yang , Z. Cui , X. F. Zhang , Y. P. Li , Eur. Polym. J. 2021, 143, 110179.

[58] A. N. Hasanah , N. Safitri , A. Zulfa , N. Neli , D. Rahayu , Molecules 2021, 26, 5612. 34577083 10.3390/molecules26185612 PMC8470890

[59] S. Bhogal , K. Kaur , I. Mohiuddin , S. Kumar , J. Lee , R. J. C. Brown , K. H. Kim , A. K. Malik , Environ. Pollut. 2021, 288, 117775. 34329047 10.1016/j.envpol.2021.117775

[60] B. Sellergren , TrAC, Trends Anal. Chem. 1997, 16, 310.

[61] W. B. Ma , X. A. Du , M. M. Liu , F. F. Gao , X. L. Ma , Y. G. Li , G. Q. Guan , X. G. Hao , Chem. Eng. J. 2021, 412, 128576.

[62] Y. Yuan , Y. J. Yang , X. J. Ma , Q. H. Meng , L. L. Wang , S. Zhao , G. S. Zhu , Adv. Mater. 2018, 30, 1706507. 10.1002/adma.201706507 29423920

[63] Z. Zhang , J. H. Li , X. L. Song , J. P. Ma , L. X. Chen , RSC Adv. 2014, 4, 46444.

[64] T. Takeuchi , T. Hishiya , Org. Biomol. Chem. 2008, 6, 2459. 18600264 10.1039/b715737c

[65] M. Kaneda , M. Okano , K. Hata , T. Sado , N. Tsujimoto , E. Li , H. Sasaki , Nature 2004, 429, 900. 15215868 10.1038/nature02633

[66] A. Bossi , M. Andreoli , F. Bonini , S. Piletsky , Anal. Bioanal. Chem. 2007, 389, 447. 17410348 10.1007/s00216-007-1258-3

[67] D. W. Zhang , Y. P. Wang , W. T. Geng , H. L. Liu , Sens. Actuators, B 2019, 285, 546.

[68] X. Wang , K. Huang , H. X. Zhang , L. S. Zeng , Y. K. Zhou , T. Jing , Mater. Sci. Eng., C 2019, 105, 110141. 10.1016/j.msec.2019.110141 31546407

[69] X. P. Fu , Y. Li , S. Gao , Y. Q. Lv , J. Sep. Sci. 2021, 44, 2483. 33835702 10.1002/jssc.202100137

[70] T. Cowen , K. Karim , S. Piletsky , Anal. Chim. Acta 2016, 936, 62. 27566340 10.1016/j.aca.2016.07.027

[71] K. Karim , F. Breton , R. Rouillon , E. V. Piletska , A. Guerreiro , I. Chianella , S. A. Piletsky , Adv. Drug Delivery Rev. 2015, 57, 1795. 10.1016/j.addr.2005.07.013 16225957

[72] S. T. Wei , M. Jakusch , B. Mizaikoff , Anal. Bioanal. Chem. 2007, 389, 423. 17569033 10.1007/s00216-007-1358-0

[73] R. J. Umpleby , G. T. Rushton , R. N. Shah , A. M. Rampey , J. C. Bradshaw , J. K. Berch , K. D. Shimizu , Macromolecules 2001, 34, 8446.

[74] C. M. F. Soares , G. M. Zanin , F. F. de Moraes , O. A. A. dos Santos , H. F. de Castro , J. Inclusion Phenom. Macrocyclic Chem. 2007, 57, 79.

[75] Y. X. Cui , A. X. Su , J. Y. Feng , W. C. Dong , J. M. Li , H. Wang , X. Y. Ni , Y. Jiang , Spectrochim. Acta, Part A 2022, 264, 120293. 10.1016/j.saa.2021.120293 34455374

[76] S. C. Liu , G. H. Lu , H. X. Ou , R. N. Shi , J. M. Pan , J. Colloid Interface Sci. 2021, 601, 782. 34107316 10.1016/j.jcis.2021.05.165

[77] W. W. Ma , Y. N. An , K. H. Row , Analyst 2019, 144, 6327. 31552929 10.1039/c9an01259a

[78] İ. Meydan , M. Bilici , E. Turan , A. Zengin , Anal. Lett. 2021, 54, 1697.

[79] A. M. Fahim , E. E. A. Magd , J. Mol. Struct. 2021, 1241, 130660.

[80] B. Sellergren , K. J. Shea , J. Chromatogr. A 1993, 635, 31. 10.1016/0021-9673(93)83061-V 8275175

[81] J. X. He , H. Y. Pan , L. Xu , R. Y. Tang , J. Chem. Res. 2021, 45, 400.

[82] Y. L. Lin , Y. Liu , S. Q. Li , L. L. Rui , J. M. Ou , Q. Z. Wu , J. F. He , J. Polym. Res. 2021, 28, 179.

[83] C. Baggiani , L. Anfossi , P. Baravalle , C. Giovannoli , C. Tozzi , Anal. Chim. Acta 2015, 531, 199.

[84] D. M. Gao , Z. P. Zhang , M. H. Wu , C. G. Xie , G. J. Guan , D. P. Wang , J. Am. Chem. Soc. 2007, 129, 7859. 17550249 10.1021/ja070975k

[85] Z. H. Wang , T. Qiu , L. H. Guo , J. Ye , L. F. He , X. Y. Li , Chem. Eng. J. 2018, 332, 409.

[86] B. B. Prasad , P. K. Pathak , Anal. Chim. Acta 2017, 974, 75e86. 28535884 10.1016/j.aca.2017.04.001

[87] W. X. Liang , H. W. Hu , P. R. Guo , Y. F. Ma , P. P. Li , W. R. Zheng , M. Zhang , Polymers 2017, 9, 344.

[88] A. A. Ozcan , R. Say , A. Denizli , A. Ersoz , Anal. Chem. 2006, 78, 7253. 17037929 10.1021/ac060536o

[89] M. F. Pan , L. P. Hong , X. Q. Xie , K. X. Liu , J. Y. Yang , S. Wang , Macromol. Chem. Phys. 2021, 222, 2000222.

[90] W. Meouche , C. Branger , I. Beurroies , R. Denoyel , A. Margaillan , Macromol. Rapid Commun. 2012, 33, 928. 22351426 10.1002/marc.201200039

[91] A. G. Mayes , K. Mosbach , Anal. Chem. 1996, 68, 3769. 21619249 10.1021/ac960363a

[92] H. N. Zeng , X. Yu , J. F. Wan , X. J. Cao , Process Biochem. 2020, 94, 329.

[93] N. Perez , M. J. Whitcombe , E. N. Vulfson , J. Appl. Polym. Sci. 2000, 77, 1851.

[94] K. C. Hua , L. Zhang , Z. H. Zhang , Y. Guo , T. Y. Guo , Acta Biomater. 2011, 7, 3086. 21605708 10.1016/j.actbio.2011.05.006

[95] H. Sanbe , J. Haginaka , Analyst 2003, 128, 593. 12866873 10.1039/b301257n

[96] S. Fauziah , A. M. G. Ma , N. H. Soekamto , P. Taba , A. Sapar , Egypt. J. Chem. 2021, 64, 2385.

[97] S. Masumoto , Y. Nakamura , J. Haginaka , J. Pharm. Biomed. Anal. 2021, 205, 114294. 34375783 10.1016/j.jpba.2021.114294

[98] H. T. Nguyen , N. T. V. Bui , W. G. Kanhounnon , K. L. V. Huynh , T. V. A. Nguyen , H. M. Nguyen , M. H. Do , M. Badawi , U. D. Thach , RSC Adv. 2021, 11, 34281. 35497320 10.1039/d1ra05505d PMC9042346

[99] Y. P. Qin , H. Y. Wang , X. W. He , W. Y. Li , Y. K. Zhang , Talanta 2018, 185, 620. 29759250 10.1016/j.talanta.2018.03.082

[100] C. X. Lu , Z. G. Tang , C. B. Liu , X. M. Ma , J. Sep. Sci. 2018, 41, 3496. 30027558 10.1002/jssc.201800474

[101] M. Zourob , S. Mohr , A. G. Mayes , A. Macaskill , N. Perez‐Moral , P. R. Fielden , N. J. Goddard , Lab Chip 2006, 6, 296. 16450041 10.1039/b513195b

[102] K. Yoshimatsu , K. Reimhult , A. Krozer , K. Mosbacha , K. Sode , L. Ye , Anal. Chim. Acta 2007, 584, 112. 17386593 10.1016/j.aca.2006.11.004

[103] A. Beltran , R. M. Marce , P. A. G. Cormack , F. Borrull , J. Chromatogr. A 2009, 1216, 2248. 19181320 10.1016/j.chroma.2009.01.024

[104] W. Liu , B. Wang , J. Appl. Polym. Sci. 2009, 113, 1125.

[105] Y. G. Feng , Q. Liu , L. F. Ye , Q. Z. Wu , J. F. He , J. Sep. Sci. 2017, 40, 971. 28012243 10.1002/jssc.201601011

[106] G. Kotan , ECS J. Solid State Sci. Technol. 2021, 10, 017003.

[107] F. Zhou , Z. J. Zheng , B. Yu , W. M. Liu , W. T. S. Huck , J. Am. Chem. Soc. 2006, 128, 16253. 17165779 10.1021/ja0654377

[108] X. X. Li , J. M. Pan , J. D. Dai , X. H. Dai , H. X. Ou , L. C. Xu , C. X. Li , R. X. Zhang , J. Sep. Sci. 2012, 35, 2787. 22997140 10.1002/jssc.201200397

[109] G. H. Cheng , X. Li , X. Li , J. F. Chen , Y. L. Li , G. Q. Zhao , G. F. Zhu , J. Hazard. Mater. 2022, 423, 127087. 34523475 10.1016/j.jhazmat.2021.127087

[110] Y. Cui , C. Tao , S. P. Zheng , Q. He , S. F. Ai , J. B. Li , Macromol. Rapid Commun. 2005, 26, 1552.

[111] X. H. Zhu , H. Li , H. Liu , W. Peng , S. A. Zhong , Y. Wang , J. Sep. Sci. 2016, 39, 2431. 27121654 10.1002/jssc.201600168

[112] Z. G. Tang , C. B. Liu , J. Wang , H. M. Li , Y. Ji , G. H. Wang , C. X. Lu , J. Sep. Sci. 2016, 39, 1592. 27106769 10.1002/jssc.201501313

[113] D. Cai , L. Ren , H. Z. Zhao , C. J. Xu , L. Zhang , Y. Yu , H. Z. Wang , Y. C. Lan , M. F. Roberts , J. H. Chuang , M. J. Naughton , Z. F. Ren , T. C. Chiles , Nat. Nanotechnol. 2010, 5, 597. 20581835 10.1038/nnano.2010.114 PMC3064708

[114] M. Arabi , A. Ostovan , J. H. Li , X. Y. Wang , Z. Y. Zhang , J. Choo , L. X. Chen , Adv. Mater. 2021, 33, 2100543. 10.1002/adma.202100543 34145950

[115] L. Pasquardini , A. M. Bossi , Anal. Bioanal. Chem. 2021, 413, 6101. 34018035 10.1007/s00216-021-03409-1 PMC8440283

[116] F. Y. Cui , Z. R. Zhou , H. S. Zhou , Sensors 2020, 20, 996.

[117] J. J. Zhou , Y. F. Wang , Y. Ma , B. L. Zhang , Q. Y. Zhang , Appl. Surf. Sci. 2019, 486, 265.

[118] J. M. Pan , W. Chen , Y. Ma , G. Q. Pan , Chem. Soc. Rev. 2018, 47, 5574. 29876564 10.1039/c7cs00854f

[119] W. Bai , D. A. Spivak , Angew. Chem., Int. Ed. 2014, 53, 2095. 10.1002/anie.201309462 24453117

[120] M. F. Jia , Z. Zhang , J. H. Li , X. Ma , L. X. Chen , X. B. Yang , TrAC, Trends Anal. Chem. 2018, 106, 190e201.

[121] J. X. Liu , Q. L. Deng , D. Y. Tao , K. G. Yang , L. H. Zhang , Z. Liang , Y. K. Zhang , Sci. Rep. 2014, 4, 5487. 24976158 10.1038/srep05487 PMC4074782

[122] E. Turan , A. Zengin , Z. Suludere , N. O. Kalkan , U. Tamer , Talanta 2022, 237, 122926. 34736663 10.1016/j.talanta.2021.122926

[123] Y. Q. Xu , T. Huang , B. Hu , M. J. Meng , Y. S. Yan , Microchem. J. 2022, 172, 106899.

[124] X. Wang , L. Y. Wang , X. W. He , Y. K. Zhang , L. X. Chen , Talanta 2009, 78, 327. 19203590 10.1016/j.talanta.2008.11.024

[125] J. Q. Fu , L. X. Chen , J. H. Li , Z. Zhang , J. Mater. Chem. A 2015, 3,13598.

[126] M. Hussain , H. Northoff , F. K. Gehring , Talanta 2016, 147, 1. 26592569 10.1016/j.talanta.2015.09.027

[127] C. G. Xie , Z. P. Zhang , D. P. Wang , G. J. Guan , D. M. Gao , J. H. Liu , Anal. Chem. 2006, 78, 8339. 17165825 10.1021/ac0615044

[128] M. H. Lee , K. T. Liu , J. L. Thomas , Z. L. Su , D. O'Hare , T. van Wuellen , J. M. Chamarro , S. Bolognin , S. C. Luo , J. C. Schwamborn , H. Y. Lin , ACS Appl. Nano Mater. 2020, 3, 8027.

[129] P. Ghaffari‐Bohlouli , P. Zahedi , M. Shahrousvand , Int. J. Biol. Macromol. 2020, 165, 2363. 33091473 10.1016/j.ijbiomac.2020.10.078

[130] K. Hasegawa , S. Tanaka , I. Bataev , D. Inao , M. Nishi , A. Kubota , K. Hokamoto , Materials 2022, 15, 1727. 35268959 10.3390/ma15051727 PMC8911162

[131] G. Ciardelli , B. Cioni , C. Cristallini , N. Barbani , D. Silvestri , P. Giusti , Biosens. Bioelectron. 2004, 20, 1083. 15556352 10.1016/j.bios.2004.06.028

[132] S. E. Diltemi , R. Say , S. Büyüktiryaki , D. Hür , A. Denizli , A. Ersöz , Talanta 2008, 75, 890. 18585161 10.1016/j.talanta.2007.12.036

[133] L. M. Chang , Y. Li , J. Chu , J. Y. Qi , X. Li , Anal. Chim. Acta 2010, 680, 65. 20969993 10.1016/j.aca.2010.09.017

[134] A. Gültekin , A. Ersöz , D. Hür , N. Y. Sarıözlü , A. Denizli , R. Say , Appl. Surf. Sci. 2009, 256, 142.

[135] M. Titirici , B. Sellergren , Chem. Mater. 2006, 18, 1773.

[136] D. L. Deng , Y. N. He , M. Y. Li , L. D. Huang , J. Z. Zhang , Environ. Sci. Pollut. Res. 2021, 28, 5966. 10.1007/s11356-020-10970-2 32981015

[137] K. Sreenivasan , R. Sivakumar , J. Appl. Polym. Sci. 1999, 71, 1823.

[138] A. Venkatesh , N. Chopra , R. J. Krupadam , Environ. Sci. Pollut. Res. 2014, 21, 6603. 10.1007/s11356-014-2566-8 24499987

[139] X. L. Song , J. H. Li , S. F. Xu , R. J. Ying , J. P. Ma , C. Y. Liao , D. Y. Liu , J. B. Yu , L. X. Chen , Talanta 2012, 99, 75. 22967524 10.1016/j.talanta.2012.04.065

[140] E. Battista , P. L. Scognamiglio , N. D. Luise , U. Raucci , G. Donati , N. Rega , P. A. Nettiabd , F. Causa , J. Mater. Chem. B 2018, 6, 1207. 32254181 10.1039/c7tb03107f

[141] O. Ramstrom , L. Andersson , K. Mosbach , J. Org. Chem. 1993, 58, 7562.

[142] U. Athikomrattanakul , N. Gajovic‐Eichelmann , F. W. Scheller , Anal. Chem. 2011, 83, 7704. 21958006 10.1021/ac201099h

[143] X. Q. Cai , J, H. , Li, Z. Z. , F. F. Yang , R. C. Dong , L. X. Chen , ACS Appl. Mater. Interfaces 2014, 6, 305. 24344795 10.1021/am4042405

[144] Y. C. Li , Y. S. Zhang , A. Akpek , S. R. Shin , A. Khademhosseini , Biofabrication 2017, 9, 012001. 10.1088/1758-5090/9/1/012001 27910820

[145] J. X. Huang , C. D. Luo , W. B. Li , Y. Li , Y. S. Zhang , J. H. Zhou , Q. Jiang , J. Mater. Chem. B 2015, 3, 4530. 32262397 10.1039/c5tb00263j

[146] C. S. Zhao , S. Q. Nie , M. Tang , S. D. Sun , Prog. Polym. Sci. 2011, 36, 1499.

[147] W. C. Li , X. B. Liu , Z. S. Deng , Y. T. Chen , Q. Q. Yu , W. Tang , T. L. Sun , Y. S. Zhang , K. Yue , Adv. Mater. 2019, 31, 1904732. 10.1002/adma.201904732 31602727

[148] S. Ambrosinia , M. Serraa , S. Shindeb , B. Sellergrenb , E. D. Lorenzi , J. Chromatogr. A 2011, 1218, 6961. 21871628 10.1016/j.chroma.2011.07.104

[149] Y. Kanekiyo , R. Naganawa , H. Tao , Angew. Chem., Int. Ed. 2003, 42, 3014. 10.1002/anie.200351381 12851958

[150] K. Çetin , H. Alkan , N. Bereli , A. Denizli , J. Macromol. Sci., Part A: Pure Appl.Chem. 2017, 54, 502.

[151] H. J. Liu , R. F. Han , M. Feng , J. F. Gao , Y. Long , Z. Zhao , Y. Wang , H. F. Mi , J. Sep. Sci. 2010, 33, 1856. 20449840 10.1002/jssc.201000038

[152] Z. Y. Chen , L. Xu , Y. Liang , M. P. Zhao , Adv. Mater. 2010, 22, 1488. 20437497 10.1002/adma.200903122

[153] C. Y. Wang , A. Javadi , M. Ghaffari , S. Q. Gong , Biomaterials 2010, 31, 4944. 20346500 10.1016/j.biomaterials.2010.02.073

[154] Z. Zhao , Y. Teng , G. L. Xu , T. T. Zhang , X. W. Kan , Anal. Lett. 2013, 46, 2180.

[155] T. Guo , Q. L. Deng , G. Z. Fang , D. H. Gu , Y. K. Yang , S. Wang , Biosens. Bioelectron. 2016, 79, 341. 26722764 10.1016/j.bios.2015.12.040

[156] T. Kubo , K. Koterasawa , T. Naito , K. Otsuka , Microporous Mesoporous Mater. 2015, 218, 112.

[157] W. N. Xing , L. Ni , X. L. Liu , Y. Y. Luo , Z. Y. Lu , Y. S. Yan , P. W. Huo , RSC Adv. 2013, 3, 26334.

[158] Z. D. Hua , Z. Y. Chen , Y. Z. Li , M. P. Zhao , Langmuir 2008, 24, 5773. 18459753 10.1021/la703963f

[159] L. Qin , X. W. He , W. Zhang , W. Y. Li , Y. K. Zhang , Anal. Chem. 2009, 81, 7206. 19655785 10.1021/ac900676t

[160] T. Zhang , Y. T. Qin , T. W. Tan , Y. Q. Lv , Part. Part. Syst. Charact. 2018, 35, 1800390.

[161] X. M. He , F. Gao , G. L. Tu , D. G. Hasko , S. Hüttner , N. C. Greenham , U. Steiner , R. H. Friend , W. T. S. Huck , Adv. Funct. Mater. 2011, 21, 139.

[162] C. B. Gao , F. L. Lyu , Y. D. Yin , Chem. Rev. 2021,121, 834. 32585087 10.1021/acs.chemrev.0c00237

[163] Y. S. Zhao , C. Y. Lo , L. C. Ruan , C. H. Pi , C. Kim , Y. Alsaid , I. Frenkel , R. Rico , T. C. Tsao , X. M. He , Sci. Robot. 2021, 6, eabd5483. 34043561 10.1126/scirobotics.abd5483

[164] A. Goulet‐Hanssens , C. J. Barrett , J. Polym. Sci., Part A: Polym. Chem. 2013, 51, 3058.

[165] T. Sajini , R. Thomasa , B. Mathew , Polymer 2019, 173, 127.

[166] H. Liu , Z. W. Deng , J. Q. Bu , Y. S. Zhang , Z. M. Zhang , Y. He , T. H. Li , P. R. Gao , Y. J. Yang , S. A. Zhong , Colloids Surf., B 2021, 208, 112126. 10.1016/j.colsurfb.2021.112126 34600360

[167] C. B. Gong , K. L. Wong , M. H. W. Lam , Chem. Mater. 2008, 20, 1353.

[168] N. Li , Q. Zhang , J. Liu , J. B. Joo , A. Lee , Y. Gan , Y. D. Yin , Chem. Commun. 2013, 49, 5135. 10.1039/c3cc41456f 23515396

[169] Q. Zhang , M. Janner , L. He , M. S. Wang , Y. X. Hu , Y. Lu , Y. D. Yin , Nano Lett. 2013, 13, 1770. 23464735 10.1021/nl400351k

[170] G. S. Jiang , S. A. Zhong , L. Chen , I. Blakey , A. Whitaker , Radiat. Phys. Chem. 2011, 80, 130.

[171] M. Dinc , C. Esen , B. Mizaikoff , TrAC, Trends Anal. Chem. 2019, 114, 202.

[172] T. Jing , H. R. Du , Q. Dai , H. Xia , J. W. Niu , Q. L. Hao , S. R. Mei , Y. K. Zhou , Biosens. Bioelectron. 2010, 26, 301. 20829022 10.1016/j.bios.2010.08.044

[173] R. J. Ansell , K. Mosbach , Analyst 1998, 123, 1611. 9830174 10.1039/a801903g

[174] Y. Zhang , R. J. Liu , Y. L. Hu , G. K. Li , Anal. Chem. 2009, 81, 967. 19178336 10.1021/ac8018262

[175] C. Giovannoli , C. Passini , F. D. Nardo , L. Anfossi , C. Baggiani , I. A. Nicholls , Polymers 2018, 10, 192. 10.3390/polym10020192 PMC6415351 30966228

[176] S. Boonpangrak , M. J. Whitcombe , V. Prachayasittiku , K. Mosbach , L. Ye , Biosens. Bioelectron. 2006, 22, 349. 16725319 10.1016/j.bios.2006.04.014

[177] S. Sasaki , T. Ooya , Y. Kitayama , T. Takeuchi , J. Biosci. Bioeng. 2015, 119, 200. 25060727 10.1016/j.jbiosc.2014.06.019

[178] S. Beyazit , B. T. S. Bui , K. Haupt , C. Gonzato , Prog. Polym. Sci. 2016, 62, 1.

[179] E. Y. Kozhunova , A. V. Plutalova , E. V. Chernikova , Polymers 2022, 14, 555. 35160544 10.3390/polym14030555 PMC8838437

[180] H. J. Wang , W. H. Zhou , X. F. Yin , Z. X. Zhuang , H. H. Yang , X. R. Wang , J. Am. Chem. Soc. 2006, 128, 15954. 17165706 10.1021/ja065116v

[181] Z. R. Li , J. X. Wang , Y. L. Song , Particuology 2011, 9, 559.

[182] W. H. Jiang , G. J. Wang , Y. N. He , X. G. Wang , Y. L. An , Y. L. Song , L. Jiang , Chem. Commun. 2005, 28, 3550. 10.1039/b504479k 16010320

[183] Z. Li , J. F. Ding , M. Day , Y. Tao , Macromolecules 2006, 39, 2629.

[184] Z. Y. Chen , H. G. Cui , K. Hales , Z. B. Li , K. Qi , D. J. Pochan , K. L. Wooley , J. Am. Chem. Soc. 2005, 127, 8592. 15954754 10.1021/ja050290p

[185] R. Randriantsilefisoa , C. X. Nie , B. Parshad , Y. W. Pan , S. Bhatia , R. Haag , Chem. Commun. 2020, 56, 3547. 10.1039/c9cc09069j 32104840

[186] J. M. Pan , B. Wang , J. D. Dai , X. H. Dai , H. Hang , H. X. Ou , Y. S. Yan , J. Mater. Chem. 2012, 22, 3360.

[187] Y. Ma , Y. Zhang , M. Zhao , X. Z. Guo , H. Q. Zhang , Chem. Commun. 2012, 48, 6217. 10.1039/c2cc31932b 22555156

[188] A. S. Tajani , V. Soheili , F. Moosavi , R. Ghodsi , T. Alizadeh , B. S. F. Bazzaz , Anal. Chim. Acta 2022, 1199, 339574. 35227378 10.1016/j.aca.2022.339574

[189] M. M. Yu , H. X. Li , J. Y. Xie , Y. Xu , X. Q. Lu , Talanta 2022, 236, 122875. 34635255 10.1016/j.talanta.2021.122875

[190] X. P. Hu , Y. D. Xia , Y. W. Liu , F. Q. Zhao , B. Z. Zeng , Microchim. Acta 2021, 188, 148.

[191] J. Matsui , K. Fujiwara , T. Takeuchi , Anal. Chem. 2000, 72, 1810. 10784146 10.1021/ac9911950

[192] S. F. Xu , H. Z. Lu , J. H. Li , X. L. Song , A. X. Wang , L. X. Chen , S. B. Han , ACS Appl. Mater. Interfaces 2013, 5, 8146. 23876063 10.1021/am4022076

[193] B. Sellergren , Anal. Chem. 1994, 66, 1578.

[194] J. Haginaka , J. Sep. Sci. 2009, 32, 1548. 19472278 10.1002/jssc.200900085

[195] M. Irshad , N. Iqbal , A. Mujahid , A. Afzal , T. Hussain , A. Sharif , E. Ahmad , M. M. Athar , Nanomaterials 2013, 3, 615. 28348356 10.3390/nano3040615 PMC5304596

[196] L. Schweitz , L. I. Andersson , S. Nilsson , Chromatographia 1999, 49, S93.

[197] X. B. Luo , W. P. Zhong , J. M. Luo , L. X. Yang , J. Long , B. Guo , S. L. Luo , J. Colloid Interface Sci. 2017, 492, 146. 28086117 10.1016/j.jcis.2016.12.065

[198] H. Hashemi‐Moghaddam , M. Hosseni , M. Mohammadhosseini , Sep. Sci. Technol. 2017, 52, 1826.

[199] K. Huang , X. Wang , H. X. Zhang , L. S. Zeng , X. Zhang , B. M. Wang , Y. K. Zhou , T. Jing , Environ. Sci. Technol. 2020, 54, 5437. 32252528 10.1021/acs.est.9b05761

[200] A. Machyˇnáková , I. Lhotská , K. Hroboˇnová , D. Satínsk´ , J. Pharm. Biomed. Anal. 2017, 145, 144. 28666160 10.1016/j.jpba.2017.06.033

[201] J. C. Xie , L. L. Zhu , X. J. Xu , Anal. Chem. 2002, 74, 2352. 12038761 10.1021/ac015755i

[202] M. Xue , Y. Wang , Z. H. Meng , W. B. Zhang , Y. Wu , S. K. Jiang , J. Liq. Chromatogr. Relat. Technol. 2013, 36, 2677.

[203] F. Yang , R. R. Wang , G. S. Na , Q. L. Yan , Z. S. Lin , Z. F. Zhang , Anal. Bioanal. Chem. 2018, 410, 1845. 29313078 10.1007/s00216-017-0843-3

[204] J. Y. Gua , H. Zhang , G. Yuan , L. R. Chen , X. J. Xu , J. Chromatogr. A 2011, 1218, 8150. 21982996 10.1016/j.chroma.2011.09.019

[205] J. X. He , G. Z. Fang , Q. L. Deng , S. Wang , Anal. Chim. Acta 2011, 692, 57. 21501712 10.1016/j.aca.2011.02.056

[206] S. Farooq , H. Y. Wu , J. Y. Nie , S. Ahmad , I. Muhammad , M. Zeeshan , R. Khan , M. Asim , Sci. Total Environ. 2022, 804, 150293. 34798762 10.1016/j.scitotenv.2021.150293

[207] G. J. Maranata , N. O. Surya , A. N. Hasanah , Heliyon 2021, 7, e05934. 33553728 10.1016/j.heliyon.2021.e05934 PMC7848654

[208] L. M. Madikizela , P. N. Nomngongo , V. E. Pakade , J. Pharm. Biomed. Anal. 2021, 208, 114447. 34740088 10.1016/j.jpba.2021.114447

[209] X. C. Guo , Z. Y. Xia , H. H. Wang , W. Y. Kang , L. M. Lin , W. Q. Cao , H. W. Zhang , W. H. Zhou , Talanta 2017, 166, 101. 28213209 10.1016/j.talanta.2017.01.047

[210] S. L. Qin , L. Q. Su , P. Wang , S. Deng , J. Appl. Polym. Sci. 2015, 132, 41491.

[211] F. F. Chen , X. Y. Xie , Y. P. Shi , J. Chromatogr. A 2013, 1300, 112. 23481473 10.1016/j.chroma.2013.02.018

[212] T. Muhammad , O. Yimit , Y. Turahun , K. Muhammad , Y. Uludag , Z. K. Zhao , J. Sep. Sci. 2014, 37, 1873. 24757081 10.1002/jssc.201400211

[213] G. Z. Fang , J. J. Feng , Y. F. Yan , C. C. Liu , S. Wang , Food Anal. Methods 2014, 7, 345.

[214] X. F. Zhang , S. X. Xu , Y. Lee , S. A. Soper , Analyst 2013, 138, 2821. 23571275 10.1039/c3an00257h

[215] X. G. Hu , Y. N. Fan , Y. Zhang , G. M. Dai , Q. L. Cai , Y. J. Cao , C. J. Guo , Anal. Chim. Acta 2012, 731, 40. 22652263 10.1016/j.aca.2012.04.013

[216] X. M. Wang , P. F. Huang , X. M. Ma , X. Z. Du , X. Q. Lu , Talanta 2019, 194, 7. 30609593 10.1016/j.talanta.2018.10.027

[217] F. Tan , H. X. Zhao , X. N. Li , X. Quan , J. W. Chen , X. M. Xiang , X. Zhang , J. Chromatogr. A 2009, 1216, 5647. 19541320 10.1016/j.chroma.2009.06.007

[218] L. Xu , Z. S. Hu , R. Duan , X. Wang , Y. S. Yang , L. Y. Dong , X. H. Wang , J. Chromatogr. A 2021, 1640, 461962. 33582517 10.1016/j.chroma.2021.461962

[219] Y. L. Hu , C. Y. Song , G. K. Li , J. Chromatogr. A 2012, 1263, 21. 23022236 10.1016/j.chroma.2012.09.029

[220] R. J. Gui , H. Jin , H. J. Guo , Z. H. Wang , Biosens. Bioelectron. 2018, 100, 56. 28863325 10.1016/j.bios.2017.08.058

[221] C. Malitesta , E. Mazzotta , R. A. Picca , A. Poma , I. Chianella , S. A. Piletsky , Anal. Bioanal. Chem. 2012, 402, 1827. 21947439 10.1007/s00216-011-5405-5

[222] J. Wackerlig , P. A. Lieberzeit , Sens. Actuators, B 2015, 207, 144.

[223] S. Carrasco , V. Canalejas‐Tejero , F. Navarro‐Villoslada , C. A. Barrios , M. C. Moreno‐Bondi , J. Mater. Chem. C 2014, 2, 1400.

[224] X. A. Ton , V. Acha , K. Haupt , B. T. S. Bui , Biosens. Bioelectron. 2012, 36, 22. 22541891 10.1016/j.bios.2012.03.033

[225] X. Lu , G. B. Zhou , J. Zhang , W. Xie , Y. W. Yang , Y. B. Zeng , Z. L. Zhang , H. L. Wang , L. Lei , ACS Sens. 2020, 5, 1445. 32295340 10.1021/acssensors.0c00368

[226] P. P. Lv , D. D. Xie , Z. H. Zhang , Talanta 2018, 188, 145. 30029356 10.1016/j.talanta.2018.05.068

[227] J. L. Urraca , M. C. Moreno‐Bondi , G. Orellana , B. Sellergren , A. J. Hall , Anal. Chem. 2007, 79, 4915. 17550229 10.1021/ac070277i

[228] W. Wan , M. Biyikal , R. Wagner , B. Sellergren , K. Rurack , Angew. Chem., Int. Ed. 2013, 52, 1. 10.1002/anie.201300322 23716378

[229] A. Valero‐Navarro , A. L. Medina‐Castillo , J. F. Fernandez‐Sanchez , A. Fernández‐Gutiérrez , Biosens. Bioelectron. 2011, 26, 4520. 21641788 10.1016/j.bios.2011.05.013

[230] E. Turan , A. Zengin , Anal. Bioanal. Chem. 2020, 412, 7417. 32812120 10.1007/s00216-020-02873-5

[231] M. Díaz‐Álvarez , A. Martín‐Esteban , Biosensors 2021, 11, 79. 33805669 10.3390/bios11030079 PMC7999655

[232] R. F. Li , Y. H. Feng , G. Q. Pan , L. Liu , Sensors 2019, 19, 177.

[233] L. Tan , C. C. Kang , S. Y. Xu , Y. W. Tang , Biosens. Bioelectron. 2013, 48, 216. 23685562 10.1016/j.bios.2013.04.024

[234] M. Y. Liu , Z. Gao , Y. J. Yu , R. X. Su , R. L. Huang , W. Qi , Z. M. He , Nanoscale Res. Lett. 2018, 13, 27. 29349585 10.1186/s11671-018-2440-6 PMC5773460

[235] H. Kubo , N. Yoshioka , T. Takeuchi , Org. Lett. 2005, 7, 359. 15673239 10.1021/ol047992o

[236] Y. S. Xia , L. Song , C. Q. Zhu , Anal. Chem. 2011, 83, 1401. 21261282 10.1021/ac1028825

[237] M. Wang , X. W. Kan , Sens. Actuators, B 2020, 323, 128672.

[238] P. S. Sharma , A. Pietrzyk‐Le , F. D'Souza , W. Kutner , Anal. Bioanal. Chem. 2012, 402, 3177. 22302165 10.1007/s00216-011-5696-6 PMC3303047

[239] A. V. Soldatova , M. Ibrahim , J. S. Olson , R. S. Czernuszewicz , T. G. Spiro , J. Am. Chem. Soc. 2010, 132, 4614. 20218710 10.1021/ja906233m PMC2853766

[240] J. P. Li , Y. P. Li , Y. Zhang , G. Wei , Anal. Chem. 2012, 84, 1888. 22242638 10.1021/ac2026817

[241] Y. Liang , L. Gu , X. Q. Liu , Q. Y. Yang , H. Kajiura , Y. M. Li , T. S. Zhou , G. Y. Shi , Chem. ‐ Eur. J. 2011, 17, 5989. 21472800 10.1002/chem.201002709

[242] F. Liu , X. W. Kan , J. Electroanal. Chem. 2019, 836, 182.

[243] M. L. Yola , T. J. Eren , N. Atar , Sens. Actuators, B 2015, 210, 149.

[244] L. Y. Ma , S. S. Miao , F. F. Lu , M. S. Wu , Y. C. Lu , H. Yang , Anal. Lett. 2017, 50, 2369.

[245] N. Karimian , M. B. Gholivand , G. Malekzadeh , J. Electroanal. Chem. 2016, 771, 64.

[246] H. Yang , L. Li , Y. P. Ding , D. X. Ye , Y. Z. Wang , S. Q. Cui , L. F. Liao , Biosens. Bioelectron. 2017, 92, 748. 27825875 10.1016/j.bios.2016.09.081

[247] Y. Liu , L. H. Zhu , Y. Y. Zhang , H. Q. Tang , Sens. Actuators, B 2012, 171, 1151.

[248] H. C. Huang , C. I. Lin , A. K. Joseph , Y. D. Lee , J. Chromatogr. A 2004, 1027, 263. 14971511 10.1016/j.chroma.2003.08.106

[249] V. K. Gupta , M. L. Yola , N. Özaltınc , N. Atard , Z. Üstündăg , L. Uzun , Electrochim. Acta 2013, 112, 37.

[250] J. Luo , J. J. Cong , J. Liu , Y. H. Gao , X. Y. Liu , Anal. Chim. Acta 2015, 864, 74. 25732429 10.1016/j.aca.2015.01.037

[251] Z. Iskierko , M. Sosnowska , P. S. Sharma , T. Benincori , F. D'Souza , I. Kaminska , K. Fronc , K. Noworyta , Biosens. Bioelectron. 2015, 74, 526. 26186151 10.1016/j.bios.2015.06.073

[252] A. Hammoud , D. Chhin , D. K. Nguyen , M. Sawan , Biosens. Bioelectron. 2021, 180, 113089. 33662846 10.1016/j.bios.2021.113089

[253] L. B. Qi , R. N. Liang , W. Qin , Anal. Chem. 2020, 92, 4284. 32090538 10.1021/acs.analchem.9b04911

[254] W. Z. Xu , Y. Y. Zhang , X. F. Yin , L. M. Zhang , Y. F. Cao , X. N. Ni , W. H. Huang , Anal. Bioanal. Chem. 2021, 413, 1081. 33247340 10.1007/s00216-020-03069-7

[255] H. F. Trevizan , A. Olean‐Oliveira , C. X. Cardoso , M. F. S. Teixeira , Sens. Actuators, B 2021, 343, 130141.

[256] U. Latif , F. L. Dickert , Sensors 2011, 11, 8611. 22164094 10.3390/s110908611 PMC3231490

[257] H. P. Liao , Z. H. Zhang , H. Li , L. H. Nie , S. Z. Yao , Electrochim. Acta 2004, 49, 4101.

[258] G. Ertürk , B. Mattiasson , Sensors 2017, 17, 390. 10.3390/s17020246 PMC5336002 28134817

[259] T. A. Sergeyeva , S. A. Piletsky , A. A. Brovko , E. A. Slinchenko , L. M. Sergeeva , A. V. El'skaya , Anal. Chim. Acta 1999, 392, 105.

[260] H. M. Duan , L. L. Li , X. J. Wang , Y. H. Wang , J. B. Li , C. N. Luo , RSC Adv. 2015, 5, 18850.

[261] J. Zhou , N. Gan , F. T. Hu , T. H. Li , H. K. Zhou , X. Li , L. Zheng , Sens. Actuators, B 2013, 186, 300.

[262] X. H. Ren , X. X. Feng , X. D. Li , X. Li , Chem. Pap. 2021, 75, 6477.

[263] M. Pesavento , L. Zeni , L. D. Maria , G. Alberti , N. Cennamo , Biosensors 2021, 11, 72. 33807535 10.3390/bios11030072 PMC8001980

[264] E. L. Holthoff , D. N. Stratis‐Cullum , M. E. Hankus , Sensors 2011, 11, 2700. 22163761 10.3390/s110302700 PMC3231613

[265] H. J. Li , X. N. Wang , Z. R. Wang , J. Q. Jiang , M. B. Wei , J. H. Zheng , Y. S. Yan , C. X. Li , Dalton Trans. 2017, 46, 11282. 28805859 10.1039/c7dt02495a

[266] B. D. Gupta , A. M. Shrivastav , S. P. Usha , Sensors 2016, 16, 1381.

[267] S. Lépinay , K. Kham , M. C. Millot , B. Carbonnier , Chem. Pap. 2012, 66, 340.

[268] A. Kidakova , J. Reut , J. Rappich , A. Öpik , V. Syritski , React. Funct. Polym. 2018, 125, 47.

[269] G. Lautner , J. Kaev , J. Reut , A. Öpik , J. Rappich , V. Syritski , R. E. Gyurcsányi , Adv. Funct. Mater. 2011, 21, 591.

[270] J. Matsui , K. Akamatsu , N. Hara , D. Miyoshi , H. Nawafune , K. Tamaki , N. Sugimoto , Anal. Chem. 2005, 77, 4282. 15987138 10.1021/ac050227i

[271] S. E. Diltemiz , R. Keçili , A. Ersöz , R. Say , Sensors 2017, 17, 454. 10.3390/s17030454 PMC5375740 28245588

[272] R. Schirhagl , Anal. Chem. 2014, 86, 250. 23944653 10.1021/ac401251j

[273] G. Z. Fang , G. Y. Liu , Y. K. Yang , S. Wang , Sens. Actuators, B 2016, 230, 272.

[274] E. Yilmaz , D. Majidi , E. Ozgur , A. Denizli , Sens. Actuators, B 2015, 209, 714.

[275] M. H. Lee , J. L. Thomas , H. Y. Tseng , W. C. Lin , B. D. Liu , H. Y. Lin , ACS Appl. Mater. Interfaces 2011, 3, 3064. 21736294 10.1021/am2005724

[276] Y. J. Zhao , L. R. Shang , Y. Cheng , Z. Z. Gu , Acc. Chem. Res. 2014, 47, 3632. 25393430 10.1021/ar500317s

[277] M. J. Liu , S. T. Wang , L. Jiang , Nat. Rev. Mater. 2017, 2, 17036.

[278] Y. J. Zhao , Z. Y. Xie , H. C. Gu , C. Zhu , Z. Z. Gu , Chem. Soc. Rev. 2012, 41, 3297. 22302077 10.1039/c2cs15267c

[279] H. X. Chen , F. K. Bian , L. Y. Sun , D. G. Zhang , L. R. Shang , Y. J. Zhao , Adv. Mater. 2020, 32, 2005394.

[280] C. K. Kuşçuoğlua , H. Günerb , M. A. Söylemezc , O. Güvenc , M. Barsbay , Sens. Actuators, B 2019, 296, 126653.

[281] N. T. Greene , K. D. Shimizu , J. Am. Chem. Soc. 2005, 127, 5695. 15826210 10.1021/ja0468022

[282] Y. X. Zhang , P. Y. Zhao , L. P. Yu , Sens. Actuators, B 2013, 181, 850.

[283] C. J. Stephenson , K. D. Shimizu , Polym. Int. 2007, 56, 482.

[284] J. Hou , H. C. Zhang , Q. Yang , M. Z. Li , L. Jiang , Y. L. Song , Small 2015, 11, 2738. 25649896 10.1002/smll.201403640

[285] N. B. Shustova , A. F. Cozzoline , S. Reineke , M. Baldo , M. Dinca , J. Am. Chem. Soc. 2013, 135, 13326. 23981174 10.1021/ja407778a

[286] B. Mattiasson , M. Hedstrom , TrAC, Trends Anal. Chem. 2016, 79, 233.

[287] Y. C. Liu , Y. J. Liu , Z. M. Liu , J. P. Hill , A. Alowasheeir , Z. G. Xu , X. T. Xu , Y. Yamauchi , J. Mater. Chem. B 2021, 9, 3192. 33885623 10.1039/d1tb00091h

